# Upcycling human excrement: the gut microbiome to soil microbiome axis

**DOI:** 10.1093/ismeco/ycaf089

**Published:** 2025-05-29

**Authors:** Jeff Meilander, Chloe Herman, Andrew Manley, Georgia Augustine, Dawn Birdsell, Evan Bolyen, Kimberly R Celona, Hayden Coffey, Jill Cocking, Teddy Donoghue, Alexis Draves, Daryn Erickson, Marissa Foley, Liz Gehret, Johannah Hagen, Crystal Hepp, Parker Ingram, David John, Katarina Kadar, Paul Keim, Victoria Lloyd, Christina Osterink, Victoria Monsaint-Queeney, Diego Ramirez, Antonio Romero, Megan C Ruby, Jason W Sahl, Sydni Soloway, Nathan E Stone, Shannon Trottier, Kaleb Van Orden, Alexis Painter, Sam Wallace, Larissa Wilcox, Colin V Wood, Jaiden Yancey, J Gregory Caporaso

**Affiliations:** Center for Applied Microbiome Science, Pathogen and Microbiome Institute, Northern Arizona University, Flagstaff, AZ, United States; Department of Biological Sciences, Northern Arizona University, Flagstaff, AZ, United States; Center for Applied Microbiome Science, Pathogen and Microbiome Institute, Northern Arizona University, Flagstaff, AZ, United States; School of Informatics, Computing, and Cyber Systems, Northern Arizona University, Flagstaff, AZ, United States; Center for Applied Microbiome Science, Pathogen and Microbiome Institute, Northern Arizona University, Flagstaff, AZ, United States; Department of Biological Sciences, Northern Arizona University, Flagstaff, AZ, United States; Department of Biological Sciences, Northern Arizona University, Flagstaff, AZ, United States; Pathogen and Microbiome Institute, Northern Arizona University, Flagstaff, AZ, United States; Center for Applied Microbiome Science, Pathogen and Microbiome Institute, Northern Arizona University, Flagstaff, AZ, United States; Pathogen and Microbiome Institute, Northern Arizona University, Flagstaff, AZ, United States; Department of Biological Sciences, Northern Arizona University, Flagstaff, AZ, United States; Genetics Core Facility, Office of the Vice President for Research, Northern Arizona University, Flagstaff, AZ, United States; Department of Biological Sciences, Northern Arizona University, Flagstaff, AZ, United States; Department of Biological Sciences, Northern Arizona University, Flagstaff, AZ, United States; Department of Biological Sciences, Northern Arizona University, Flagstaff, AZ, United States; Pathogen and Microbiome Institute, Northern Arizona University, Flagstaff, AZ, United States; Pathogen and Microbiome Division, Translational Genomics Research Institute, Flagstaff, AZ, United States; Department of Biological Sciences, Northern Arizona University, Flagstaff, AZ, United States; Center for Applied Microbiome Science, Pathogen and Microbiome Institute, Northern Arizona University, Flagstaff, AZ, United States; Center for Applied Microbiome Science, Pathogen and Microbiome Institute, Northern Arizona University, Flagstaff, AZ, United States; School of Informatics, Computing, and Cyber Systems, Northern Arizona University, Flagstaff, AZ, United States; Pathogen and Microbiome Division, Translational Genomics Research Institute, Flagstaff, AZ, United States; Department of Biological Sciences, Northern Arizona University, Flagstaff, AZ, United States; Department of Biological Sciences, Northern Arizona University, Flagstaff, AZ, United States; Department of Biological Sciences, Northern Arizona University, Flagstaff, AZ, United States; Center for Applied Microbiome Science, Pathogen and Microbiome Institute, Northern Arizona University, Flagstaff, AZ, United States; Department of Biological Sciences, Northern Arizona University, Flagstaff, AZ, United States; Department of Biological Sciences, Northern Arizona University, Flagstaff, AZ, United States; Department of Biological Sciences, Northern Arizona University, Flagstaff, AZ, United States; Department of Biological Sciences, Northern Arizona University, Flagstaff, AZ, United States; Department of Biological Sciences, Northern Arizona University, Flagstaff, AZ, United States; Center for Applied Microbiome Science, Pathogen and Microbiome Institute, Northern Arizona University, Flagstaff, AZ, United States; Department of Biological Sciences, Northern Arizona University, Flagstaff, AZ, United States; Pathogen and Microbiome Division, Translational Genomics Research Institute, Flagstaff, AZ, United States; Center for Applied Microbiome Science, Pathogen and Microbiome Institute, Northern Arizona University, Flagstaff, AZ, United States; Pathogen and Microbiome Institute, Northern Arizona University, Flagstaff, AZ, United States; Department of Biological Sciences, Northern Arizona University, Flagstaff, AZ, United States; Department of Biological Sciences, Northern Arizona University, Flagstaff, AZ, United States; Department of Biological Sciences, Northern Arizona University, Flagstaff, AZ, United States; Department of Biological Sciences, Northern Arizona University, Flagstaff, AZ, United States; Department of Biological Sciences, Northern Arizona University, Flagstaff, AZ, United States; Center for Applied Microbiome Science, Pathogen and Microbiome Institute, Northern Arizona University, Flagstaff, AZ, United States; Department of Biological Sciences, Northern Arizona University, Flagstaff, AZ, United States; Center for Applied Microbiome Science, Pathogen and Microbiome Institute, Northern Arizona University, Flagstaff, AZ, United States; Department of Biological Sciences, Northern Arizona University, Flagstaff, AZ, United States; School of Informatics, Computing, and Cyber Systems, Northern Arizona University, Flagstaff, AZ, United States; Pathogen and Microbiome Division, Translational Genomics Research Institute, Flagstaff, AZ, United States

**Keywords:** human excrement, compost, 16S, amplicon, manure, biosolids, microbiome, latrines, compost toilet, soil, sustainability

## Abstract

Human excrement composting (HEC) is a sustainable strategy for human excrement (HE) management that recycles nutrients and mitigates health risks while reducing reliance on freshwater, fossil fuels, and fertilizers. A mixture of HE and bulking material was collected from 15 composting toilets and composted as 15 biological replicates in modified 19-liter buckets under mesophilic conditions with weekly sampling for one year. We hypothesized that (i) the microbiome of 1 year old compost would resemble that of a soil and/or food and landscape waste compost microbiome more closely than the original HE; and (ii) the human fecal indicators, *Escherichia coli* and *Clostridium perfringens*, would be undetectable after 52 weeks using qPCR and culturing. This investigation identified unique successional trajectories within buckets (i.e. biological replicates) and significant shifts in microbial communities around 25 weeks across buckets, with reductions in fecal-associated taxa and increases in environmental taxa indicating effective composting. We present a comprehensive microbial time series analysis of HEC and show that the initial gut-like microbiome of HEC systems transitions to a microbiome similar to soil and traditional compost but that pathogen risk assessment is important if thermophilic temperatures are not achieved. This study also produced the highest resolution composting microbiome data to date, establishing a baseline for HEC optimization and thermophilic composting studies while serving as a resource for bioprospecting for enzymes and organisms relevant to upcycling waste.

## Introduction

Traditional wastewater treatment infrastructure depletes freshwater, diverts nutrients to landfills, requires significant amounts of energy, and fails to meet the needs of 2 billion people [1] resulting in public and environmental health concerns. Human excrement (HE) management represents a global challenge in the context of exponential population growth and rapidly changing climates. Human excrement composting (HEC) utilizing composting toilets (CTs) offers low-cost alternative sanitation solutions, eliminates flushing, and reduces energy consumption while creating economic opportunities and recycling nutrients [2].

By sequencing the V4 region of the 16S rRNA gene coupled with qPCR and culturing experiments, we investigated microbial succession during 1 year of mesophilic HEC across 15 biological replicates generating the most extensive and high-resolution dataset to date along the gut microbiome to soil microbiome (gut-to-soil) axis. We hypothesized (i) the microbiome of 1 year old HEC would resemble soil and/or food and landscape waste compost (FLWC) microbiomes more closely than the original HE and (ii) human fecal indicators, *Escherichia coli* and *Clostridium perfringens*, would be undetectable after 52 weeks. Replicates were maintained independently in 19-l buckets. We therefore refer to each replicate as a “bucket” herein.

Microbial communities driving efficient composting operate within ecological niches defined by optimal ranges of variables including temperature (>55°C), carbon to nitrogen ratio (C:N 30:1), and moisture content (45%–65%) [3–5]. Optimization accelerates biodegradation of recalcitrant compounds, enhances humification, and reduces risks associated with pathogens, per- and polyfluoroalkyl substances (PFAS), pharmaceuticals, and antimicrobial resistance genes (ARG) [6–8].

**Figure 1:**
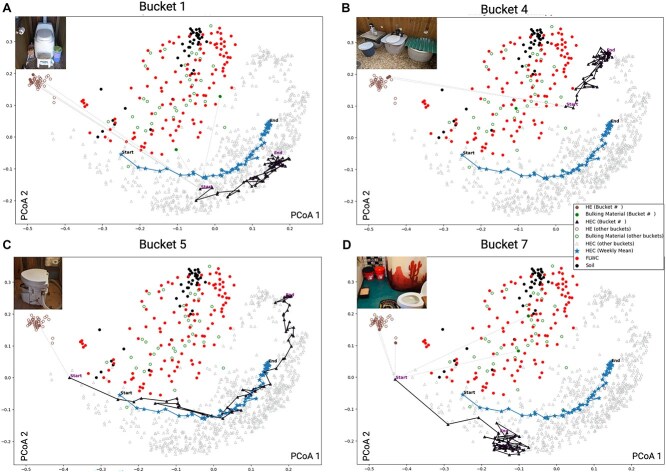
Distinct trajectories of HEC microbiomes along the gut-to-soil microbiome axis across four representative composting buckets, as visualized by two-dimensional PCoA plots. Two-dimensional principal coordinate analysis (PCoA) was applied to the unweighted UniFrac distance matrix computed between all samples including soil and food and landscape waste compost (FLWC) reference samples. The trajectory through the ordination space for individual buckets is plotted with the statistical mean of ordination results across all 15 buckets (denoted by stars connected by a line) at each composting time point—defined as the number of weeks since HEC was retrieved from the participant and actively managed (Fig. S1). Averages are not points within the ordination, but rather overlaid on the ordination plot. The average trajectory exhibits a progressive movement away from HE along Axis 1 over the course of one year, with final TPs clustering closer to reference samples. Images of the composting toilets corresponding to each bucket are included to illustrate the variability between systems. (A) Bucket 1’s transition rapidly diverges from HE and its rate of transition appears to decrease around week 11 with minimal further variation. (B) Bucket 4’s initial transition occurs rapidly, beginning and ending less similar to fecal material than average. (C) Bucket 5 begins more similar to HE and has the greatest extent of change relative to all buckets ending with a microbiome profile similar to reference samples and B4. (D) Bucket 7’s transition appears stunted, beginning and ending more similar to HE than average. These microbiome composition data do not offer sufficient resolution to provide clear identification of all pathogens or provide insights into microbial activity; future studies will address these aspects. Definitions: *Extent*—Euclidean distance between two data points in the ordination. *Rate—*extent divided by the difference in time between two data points in the ordination. A higher rate is illustrated by consecutive data points spaced farther apart, indicating rapid changes. Conversely, a lower rate is characterized by data points that remain relatively close. *Trajectory—*successional pattern of the microbiomes, illustrated by consecutive data points connected by a line on the ordination plot for each bucket. Some trajectories span the entire plot, reflecting substantial changes, while others display trajectories that cluster tightly, indicating reduced variability (i.e., high stability) in microbiome composition. Plots for all 15 replicates are presented in Fig. S2. Created in BioRender.

Buckets 1, 4, 5, 7 (B1, B4, B5, B7) are presented here as they exhibit the most distinct differences in extent, rate, and trajectory of microbiome transitions. (Fig. 1). Ordinated Unweighted UniFrac distances indicated microbiome compositions transitioning from HE toward reference samples along Axis 1, which was correlated with time (ρ = 0.431; *P* = .001).

CT design and inputs (HE and bulking material) likely influence microbiome transitions. B1, with its larger storage chamber and enhanced ventilation, may have facilitated a more rapid shift under aerobic conditions (Fig. 1A). B4’s design, an in-ground three-barrel system, includes close proximity to the soil, prolonged retention of material, and increased aeration. Biweekly auger mixing from older to fresher material inoculates fresh inputs with mature compost microbiomes, potentially accelerating their initial microbial transition (Fig. 1B). B5’s small, unventilated chamber led to HEC with persistent foul odors (atypical for well-managed CTs), initial elevated pH, and near 100% moisture, likely inducing anaerobic conditions that shaped its microbiome trajectory (Fig. 1C). Finally, B7’s stalled transition underscores the need to understand how system designs and environmental factors disrupt microbiome succession. Comparing B7 to successful transitions could reveal improvement strategies for efficiency and HEC safety. (Fig. 1D).

Although no two buckets converge on the same microbial endpoint or fully align with reference microbiomes, their trajectories shift away from HE, indicating that the material is no longer microbiologically equivalent to HE (Fig. S3). This also suggests that the composting process remains incomplete after one year and may require additional time or interventions—such as thermophilic composting—to achieve a more stable microbial state. We hypothesize that inoculants from well-functioning CTs, soils, or mature compost may accelerate the transition from fecal-like microbiomes, as observed in B4, by introducing soil and compost-associated microbes that shape composting time point 1 (TP-1) composition and influence its trajectory.

**Figure 2:**
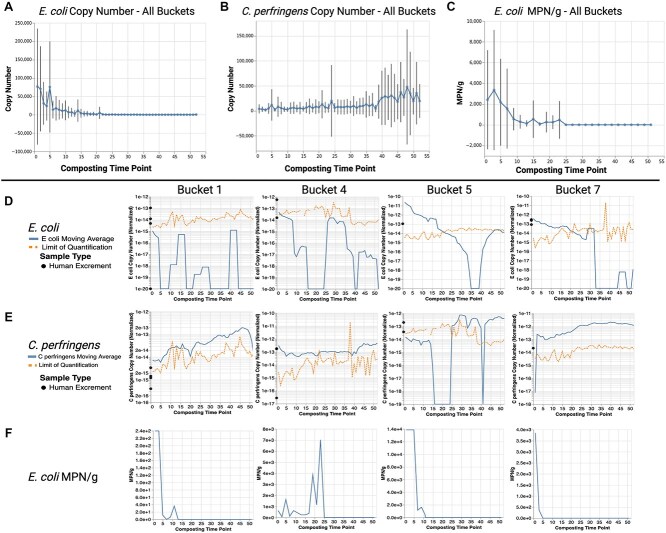
qPCR and culturing data for select buckets. (A) Mean copy number of *E. Coli* across all 16 composting buckets at each composting TP. Elevated variability during the first 15 weeks reflects heterogeneity in abundance across systems. Copy numbers declined over time, likely due to increasingly unfavorable environmental conditions for *E. Coli*. (B) Mean copy number of *C. Perfringens* across all 16 composting buckets at each composting TP. Elevated variability during the final 15 weeks reflects heterogeneity in abundance across buckets. Copy numbers increased over time, likely due to increasingly favorable environmental conditions for *C. Perfringens*. (C) Mean most probable number (MPN/g) of *E. Coli* across all 16 composting buckets at each composting TP. No culturable *E. Coli* was detected after TP-25. (A–C) Error bars represent ±1 standard deviation. Data points above the 99.9th percentile were excluded to reduce the influence of outliers, except in (C), where insufficient data precluded this filtering. (D) Copy numbers (solid line) of the uidA gene found in *E. Coli* normalized by 16S rRNA gene copy number data from the BactQuant assay (indicative of total bacterial load). By TP-52, each bucket remained below the LOQ. (E) Copy numbers (solid line) of the *C. Perfringens* group normalized by BactQuant. By TP-52, each bucket remained above the LOQ. A moving average period of four TPs was applied to (D) and (E). A pseudo-count, defined as the floor of the log10 of the minimum, minus one, was added to all data points but varied by plot because the minimum nonzero quantification varies by plot. The y-axis scale is not consistent across buckets to highlight individual variability in copy numbers. Significant fluctuations in data are attributed to sampling variability, given that TaqMan PCR analysis was conducted in triplicate. The LOQ is 304 (dashed line) normalized by BactQuant and fluctuates over time as a result. The limit of detection (LOD) of 30.4 (not displayed in the figure), was also normalized by BactQuant. Normalized data for all buckets can be found in Fig. S4 and raw results of the BactQuant assay are presented in Fig. S5. Non-normalized data for all buckets can be found in Fig. S6. (F) Biweekly samples (odd TPs) were cultured in EC broth with 4-methylumbelliferyl-β-D-glucuronide (MUG) for the detection of *E. Coli*. Data is presented linearly and the y-axis is not consistent across buckets to highlight the variability in the composting systems. Culturing data for all buckets can be found in Fig. S7. Created in BioRender.

A qPCR analysis of *E. coli* indicated a negative correlation between copy number and time (ρ = −0.49, *P* = 2.73e-48; Fig. 2A) across all buckets, with most dropping below the limit of quantification (LOQ = 304) between TPs 20–35 (Fig. S2a) and remaining below the limit of detection (LOD = 30.4). A positive correlation was observed between *C. perfringens* copy number and time (ρ = 0.28, *P* = 4.96e-16; Fig. 2B) across all buckets with many TPs remaining above or increasing beyond the LOQ over time (Fig. S2b).

Culturing *E. coli* indicated declines in all buckets (ρ = −0.546, *P* = 4.93e-31), eventually becoming undetectable by TP-25 (Fig. 2C). Detection and quantification using qPCR after TP-25 likely reflects the method’s sensitivity in detecting viable but nonculturable *E. coli* or non-viable DNA, in which the cells are no longer alive, but residual genetic material persists in the environment [9]. Due to the substantial heterogeneity among the 15 composting systems and the distinct successional trajectories observed across buckets, trends within each bucket were highly variable (Fig. 2D–F).

Small-scale composting rarely reaches thermophilic temperatures (required for proper sanitization) unless insulated or externally heated [10] and leads to variability in the composting. The persistence of fecal indicators was likely due to regrowth influenced by low temperatures, nutrient availability, or other environmental factors. While mesophilic composting can reduce pathogens, longer composting durations are recommended to enhance pathogen reduction [11].

Our primary hypothesis, that the microbiome of 1 year old HEC would resemble soil and/or FLWC microbiomes more closely than the original HE, was supported by the data, while our secondary hypothesis, that the human fecal indicators, *E. coli* and *C. perfringens*, would be undetectable after 52 weeks, was only supported by *E. coli* culturing data and not the qPCR results for either indicator.

Significant shifts in microbial communities occurred around TP-25 (Fig. S8), with reductions in fecal-associated taxa and increases in environmental taxa linked to nutrient cycling and organic matter decomposition, indicating effective composting [2, 4], (Fig. S9). Reduced rates of microbial succession by TP-52 may result from stabilization due to uniform physicochemical conditions, reduced nutrient availability, or microbial interactions [12].

Analyzing the microbiomes across all TPs in all buckets reveals variations in community composition, transition dynamics, and the extent of transition from fecal-like to soil-like microbiomes, suggesting CT inputs, design, and maintenance may impact composting efficiency. Stalled transitions might indicate improper use or suboptimal environmental conditions. Remediation with mature compost or soil might reboot the transition (Fig. S2).

Key taxa such as *Rhodanobacter, Rhodococcus, Arachidicoccus, Acinetobacter*, *Pseudomonas,* and *Hyphomicrobium* demonstrate essential roles in nutrient cycling and potentially serve as bioindicators of community succession or stabilization (Fig. S8). Furthermore, high taxonomic diversity suggests that HEC microbiomes may prove fruitful for bioprospecting for enzymes or microbial consortia useful in decomposing materials potentially harmful to human and/or environmental health (interactive taxonomic bar plots for each biological replicate can be found at DOI 10.5281/zenodo.13887457).

The presence of *E. coli* and *C. perfringens* at TP-52 aligns with results of previous studies [2, 4, 13, 14]. While not all *E. coli* and *Clostridium* species are pathogenic, risk management plans should be established, particularly while transferring material from CTs into thermophilic piles [4, 15]. Guidance from resources like *The Humanure Handbook* [16] can assist CT users in safely managing HEC.

There is an urgency to re-evaluate broad applicability of HEC amid rising challenges from climate change and population growth. Additionally, we hypothesize that investigating HEC microbiomes can discover biotechnologically relevant enzymes and organisms and support engineering of useful synthetic microbial communities. This study comprehensively assesses microbial succession during mesophilic composting of HE, addressing a key gap in understanding the gut-to-soil microbiome transition in HEC. This study also produced the highest resolution composting microbiome data to date, establishing a baseline for HEC optimization while serving as a resource for bioprospecting organisms relevant to upcycling waste.

## Supplementary Material

Edited_G2S_Supplement_5_22_25_ycaf089

fig1-legend_ycaf089

fig1_ycaf089

fig2d-legend_ycaf089

fig2e-legend_ycaf089

figS1_ycaf089

figS2_ycaf089

figS2legend_ycaf089

figS3_ycaf089

figS4_ycaf089

figS4a-legend-ecoli_ycaf089

figS4b-legend-cperf_ycaf089

figS5_ycaf089

figS6_ycaf089

figS6a-legend-ecoli_ycaf089

figS6b-legend-cperf_ycaf089

figS7_ycaf089

figS8_ycaf089

figS8legend_ycaf089

figS9_ycaf089

figS9legend_ycaf089

figS10

figS11_ycaf089

figS12_ycaf089

figS13_ycaf089

figS14_ycaf089

figS15_ycaf089

figS15legend_ycaf089

figS16_ycaf089

figS16a-S16b-legend_ycaf089

figS17_ycaf089

figS17legend_ycaf089

figS18_ycaf089

figS18legend_ycaf089

figS19_ycaf089

tableS1_ycaf089

tableS2_ycaf089

## Data Availability

Raw and processed data for this study is available in Zenodo under DOI 10.5281/zenodo.13887457.

## Code availability

Custom software was developed to create Figs 1 and 2. This code is available in a QIIME 2 “Single Use Plugin” at https://github.com/caporaso-lab/gut-to-soil-manuscript-figures, and in Zenodo under DOI 10.5281/zenodo.13887457.

## References

[1] UNICEF and WHO . State of the World’s Sanitation: An Urgent Call to Transform Sanitation for Better Health, Environments, Economies and Societies. New York, NY USA: United Nations Children’s Fund (UNICEF) and the World Health Organization, 2021.

[2] Williams LE, Kleinschmidt CE, Mecca S. Bacterial communities in the digester bed and liquid effluent of a microflush composting toilet system. *PeerJ* 2018;6:e6077. 10.7717/peerj.6077 30564526 PMC6286801

[3] Haug RT . The Practical Handbook of Compost Engineering, 2nd edn. Boca Raton, Florida: Routledge, 1993.

[4] Piceno YM, Pecora-Black G, Kramer S et al. Bacterial community structure transformed after thermophilically composting human waste in Haiti. *PLoS One* 2017;12:e0177626. 10.1371/journal.pone.0177626 28570610 PMC5453478

[5] Rynk R, Black G, Gilbert J. et al. (eds.). The Composting Handbook: A how-to and why Manual for Farm, Municipal, Institutional and Commercial Composters, 1st edn. Cambridge, Massachusetts: Academic Press, 2021.

[6] Werner KA, Poehlein A, Schneider D et al. Thermophilic composting of human feces: development of bacterial community composition and antimicrobial resistance gene pool. *Front Microbiol* 2022;13:824834. 10.3389/fmicb.2022.824834 35250940 PMC8895236

[7] Huang S, Sima M, Long Y et al. Anaerobic degradation of perfluorooctanoic acid (PFOA) in biosolids by *Acidimicrobium* sp. strain A6. *J Hazard Mater* 2022;424:127699. 10.1016/j.jhazmat.2021.127699 34799154

[8] Jonidi-Jafari A, Farzadkia M, Gholami M et al. The efficiency of removing metronidazole and ciprofloxacin antibiotics as pharmaceutical wastes during the process of composting. *Int J Environ Anal Chem* 2020;102:1–11. 10.1080/03067319.2020.1781838

[9] Oliver DM, Bird C, Burd E et al. Quantitative PCR profiling of *Escherichia coli* in livestock feces reveals increased population resilience relative to culturable counts under temperature extremes. *Environ Sci Technol* 2016;50:9497–505. 10.1021/acs.est.6b02657 27454176

[10] Niwagaba C, Nalubega M, Vinnerås B et al. Bench-scale composting of source-separated human faeces for sanitation. *Waste Manag* 2009;29:585–9. 10.1016/j.wasman.2008.06.022 18692381

[11] Holmqvist A, Stenström TA. Survival of ascaris suum ova, indicator bacteria and salmonella typhimurium phage 28b in mesophilic composting of household waste. First International Conference of Ecological Sanitation, 5th-8th November, Nanning, China, 2001;99–103.

[12] Qiu X, Zhou G, Zhang J et al. Microbial community responses to biochar addition when a green waste and manure mix are composted: a molecular ecological network analysis. *Bioresour Technol* 2019;273:666–71. 10.1016/j.biortech.2018.12.001 30528727

[13] Vinnerås B, Holmqvist A, Bagge E et al. The potential for disinfection of separated faecal matter by urea and by peracetic acid for hygienic nutrient recycling. *Bioresour Technol* 2003;89:155–61. 12699934 10.1016/s0960-8524(03)00044-0

[14] Tønner-Klank L, Møller J, Forslund A et al. Microbiological assessments of compost toilets: in situ measurements and laboratory studies on the survival of fecal microbial indicators using sentinel chambers. *Waste Manag* 2007;27:1144–54. 10.1016/j.wasman.2006.04.021 16908129

[15] Krause A, Häfner F, Augustin F et al. Qualitative risk analysis for contents of dry toilets used to produce novel recycling fertilizers. *Circ Econ Sustain* 2021;1:1107–46. 10.1007/s43615-021-00068-3 34888571 PMC8280996

[16] Jenkins J . The Humanure Handbook: A Guide to Composting Human Manure, 3rd edn. Grove City, PA USA: Joseph Jenkins, Inc, 2005.

