# Supplementary figures and images for: Upcycling human excrement: the gut microbiome to soil microbiome axis
Source: ISME Commun. 2025 May 29;5(1):ycaf089. doi: 10.1093/ismeco/ycaf089 (PMC12393218; doi:10.1093/ismeco/ycaf089)

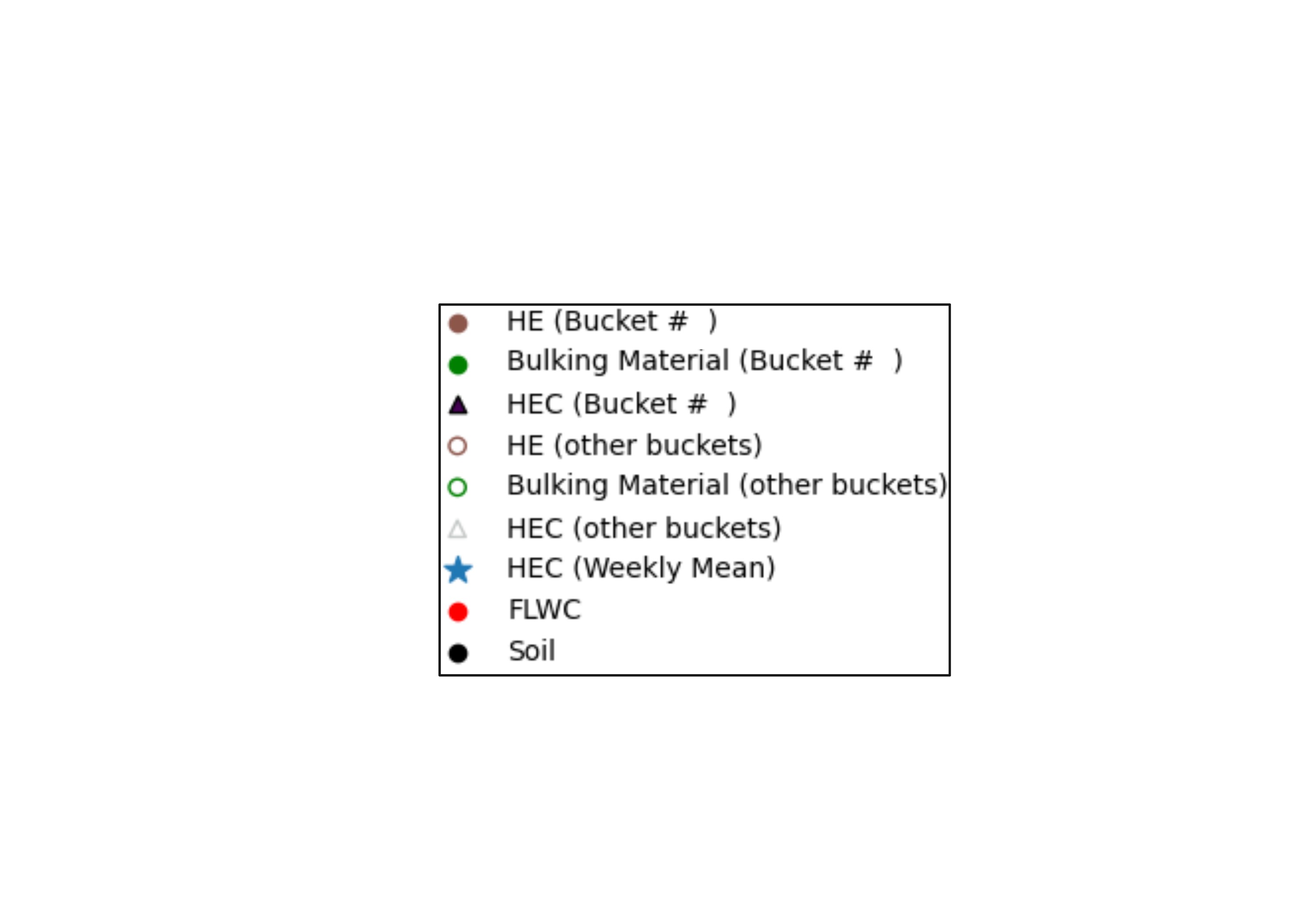

Supplement: fig1-legend_ycaf089 [file fig1-legend_ycaf089.jpeg]

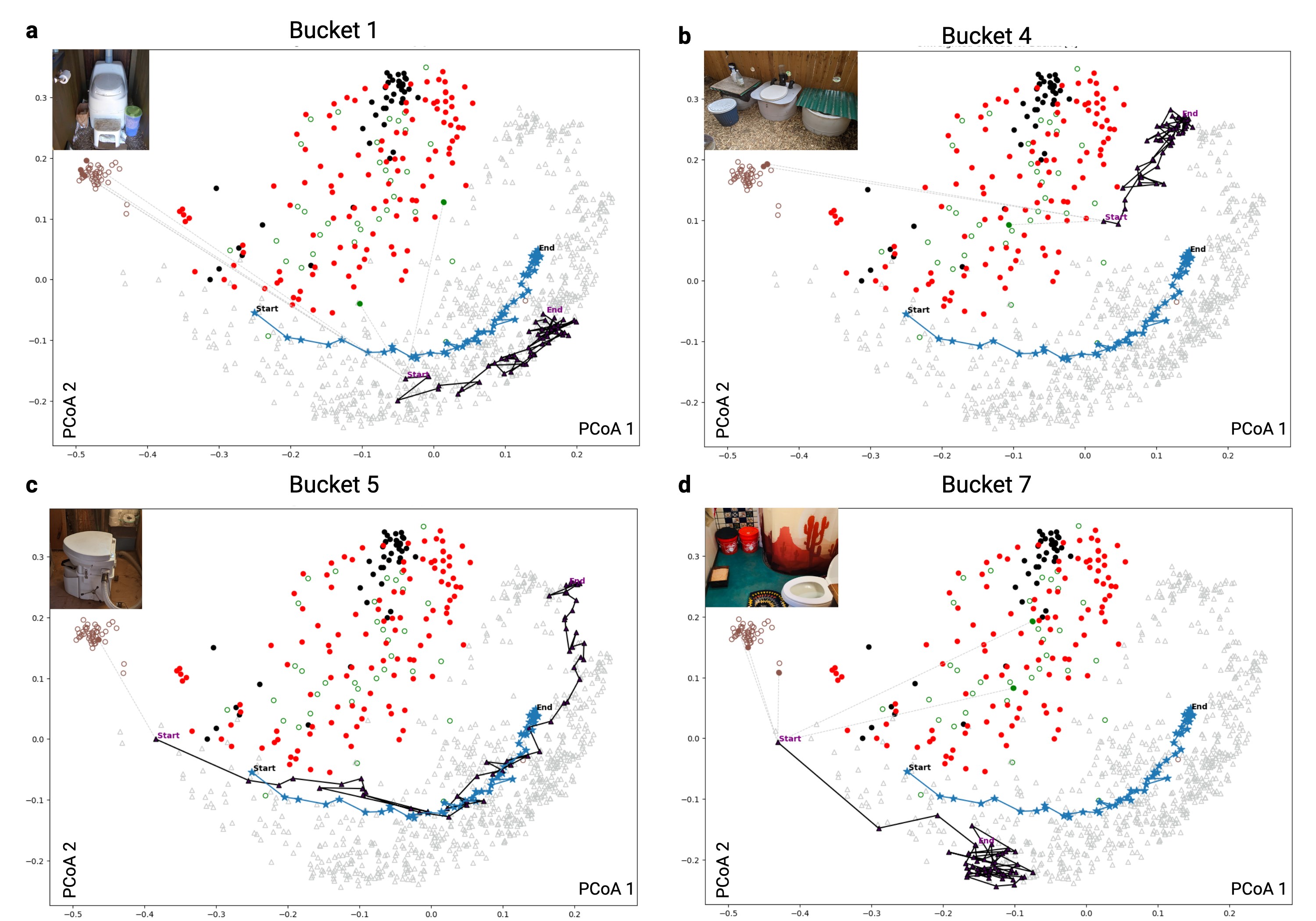

Supplement: fig1_ycaf089 [file fig1_ycaf089.jpeg]

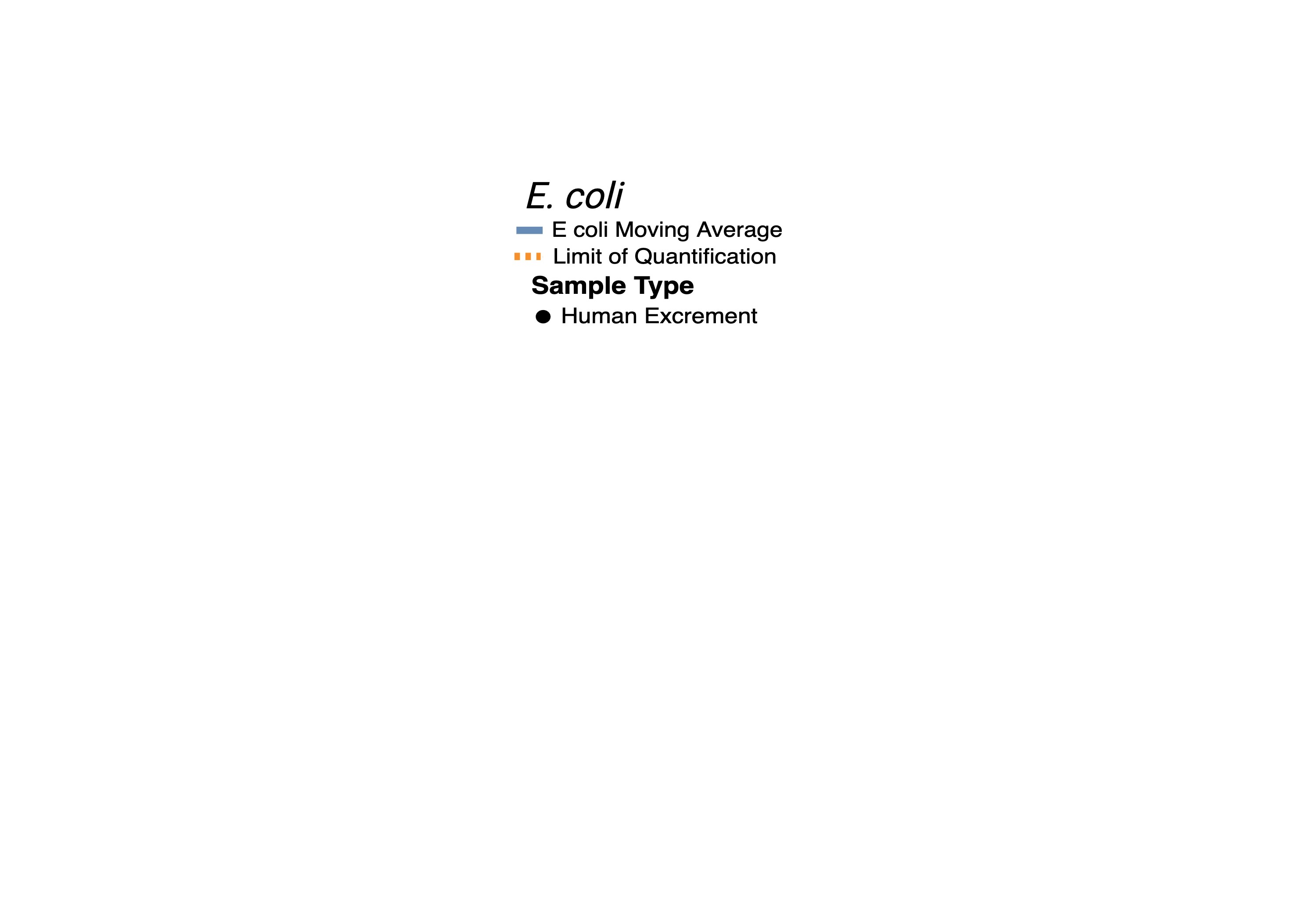

Supplement: fig2d-legend_ycaf089 [file fig2d-legend_ycaf089.jpeg]

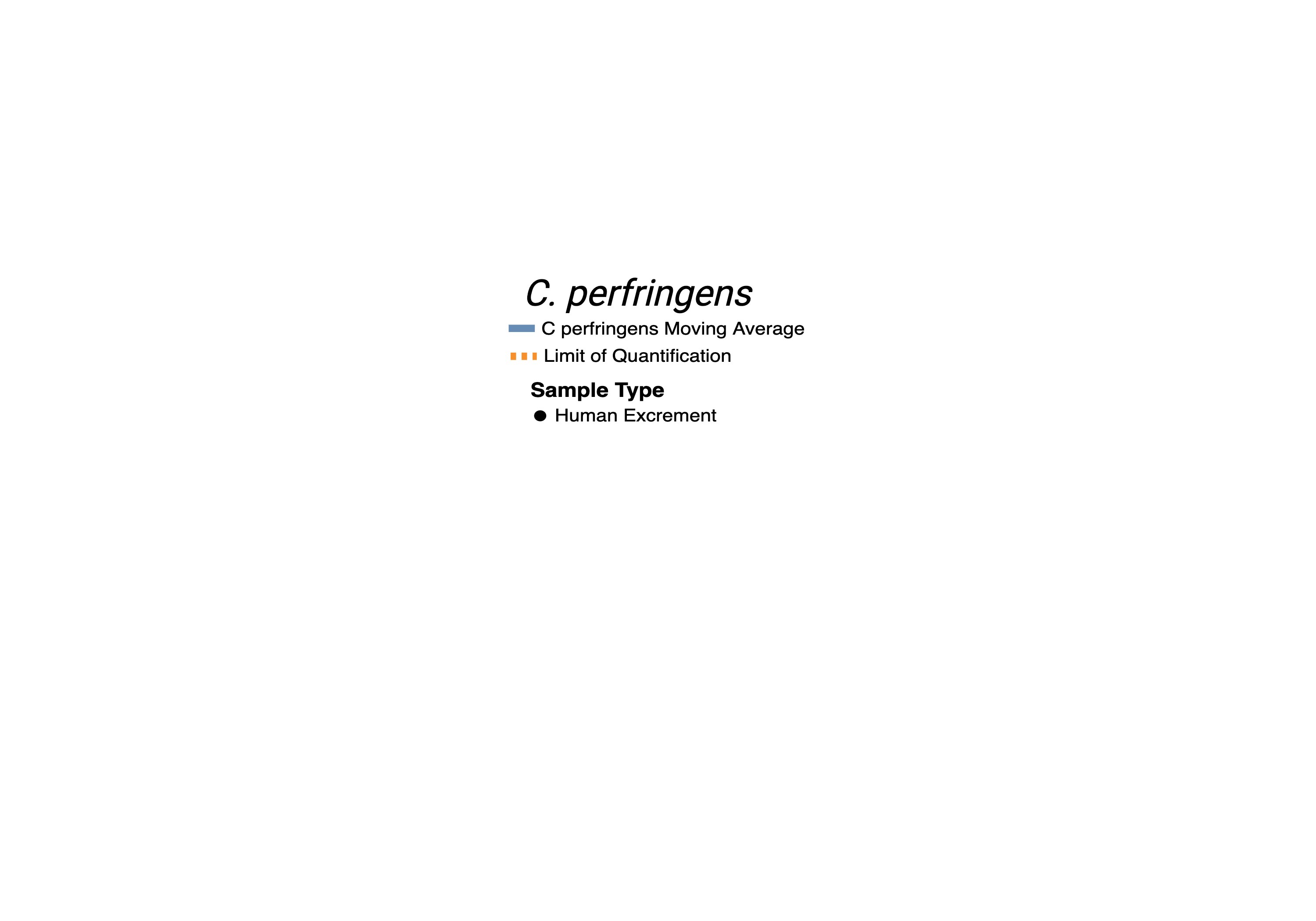

Supplement: fig2e-legend_ycaf089 [file fig2e-legend_ycaf089.jpeg]

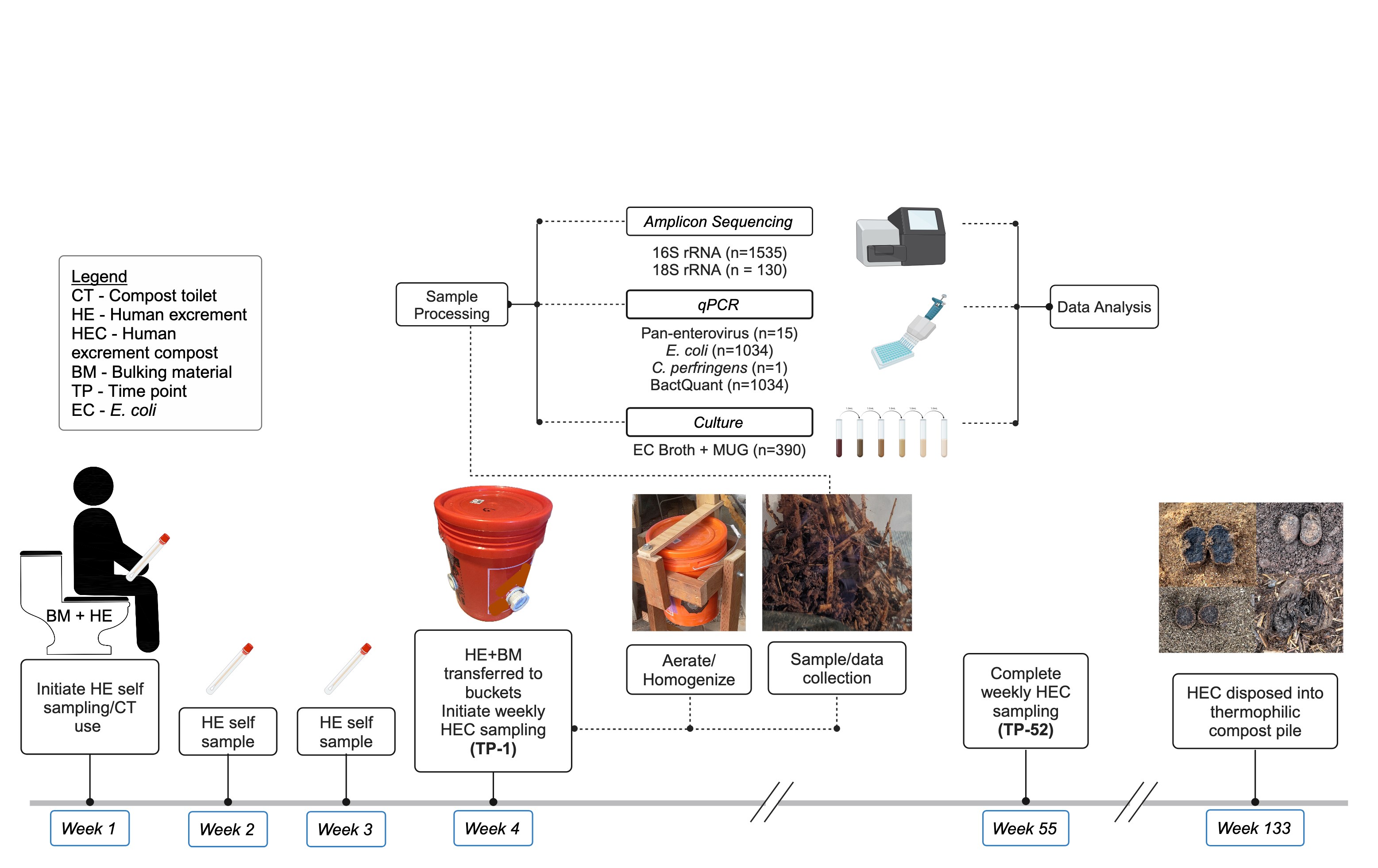

Supplement: figS1_ycaf089 [file figs1_ycaf089.jpeg]

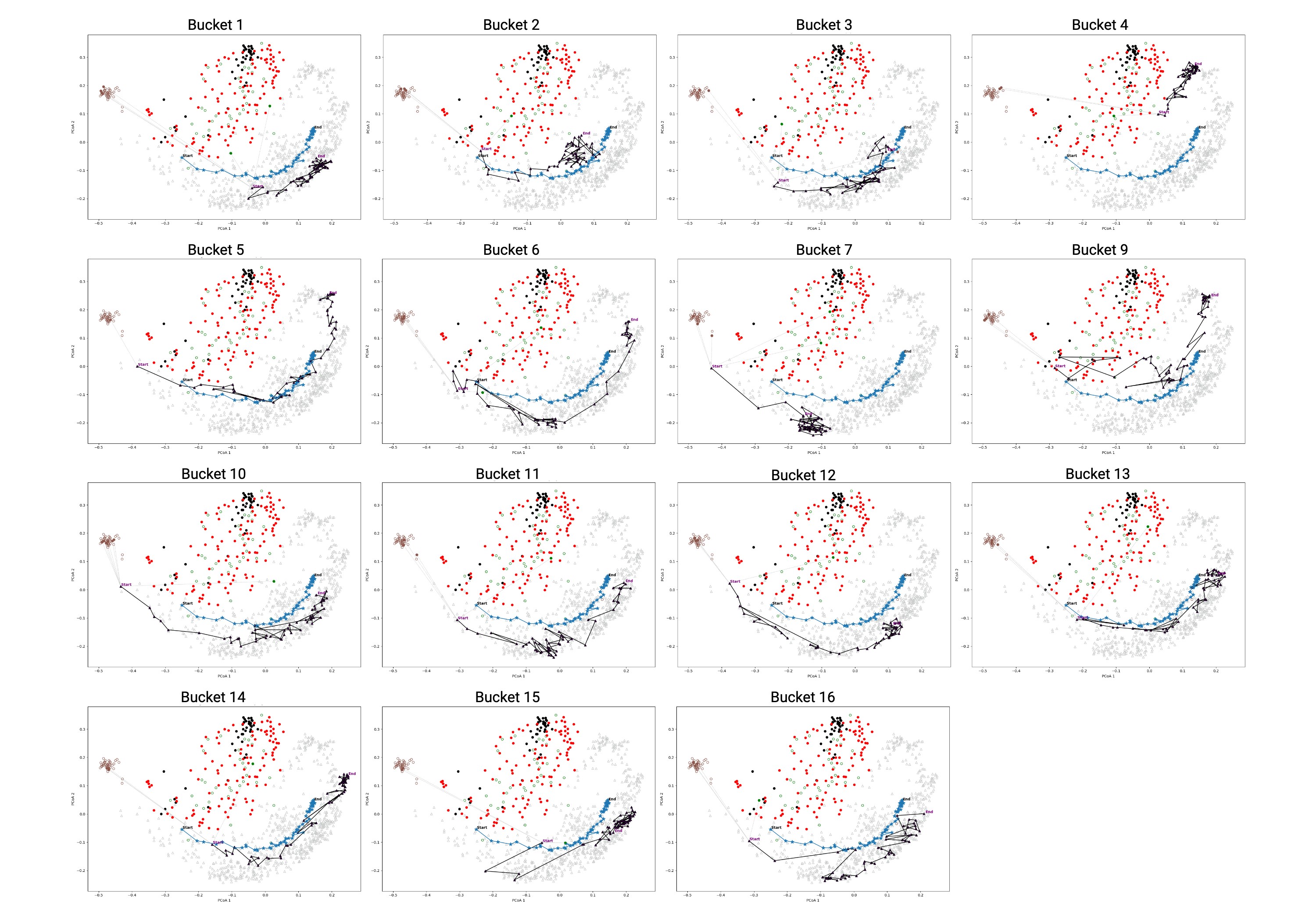

Supplement: figS2_ycaf089 [file figs2_ycaf089.jpeg]

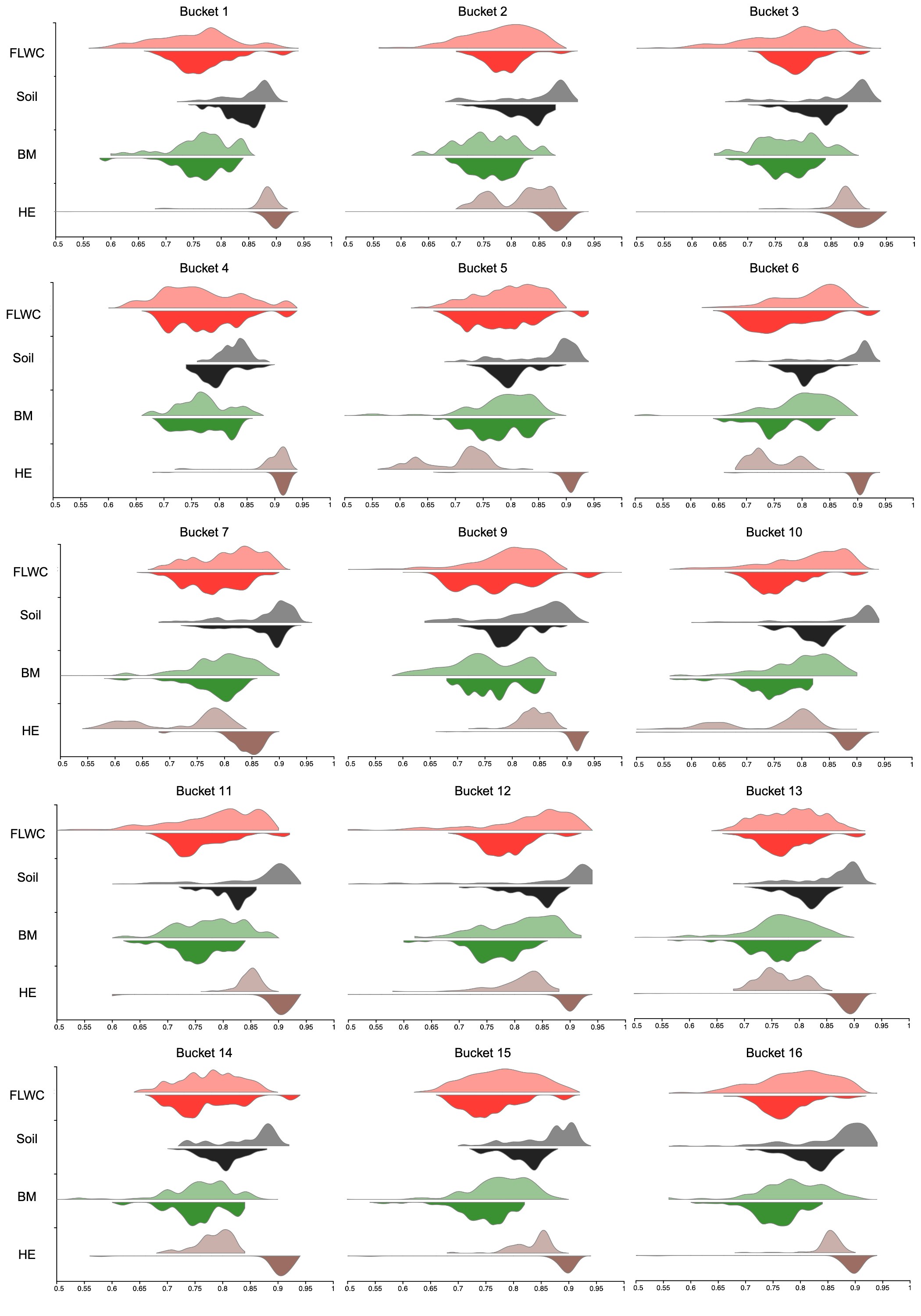

Supplement: figS3_ycaf089 [file figs3_ycaf089.jpeg]

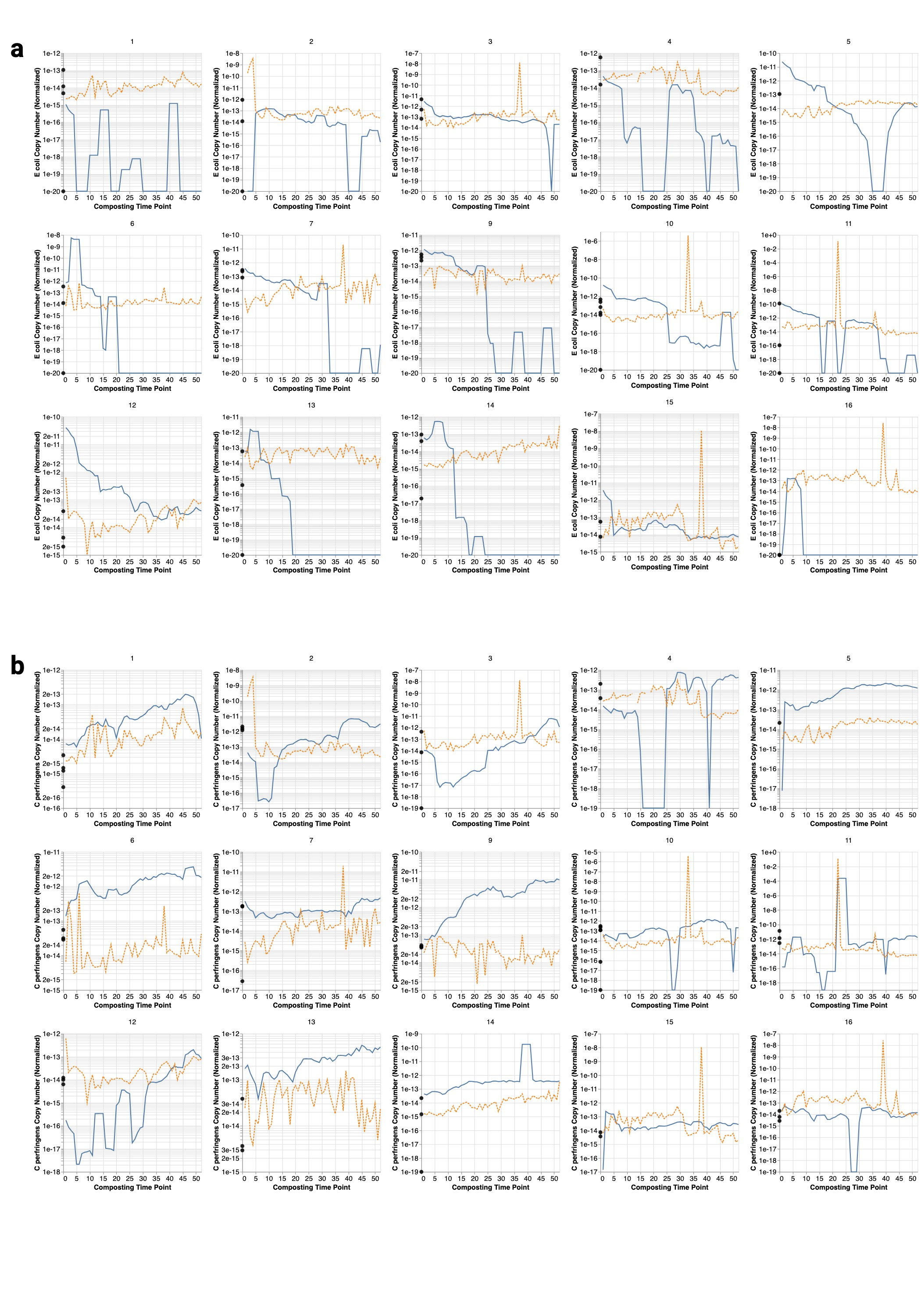

Supplement: figS4_ycaf089 [file figs4_ycaf089.jpeg]

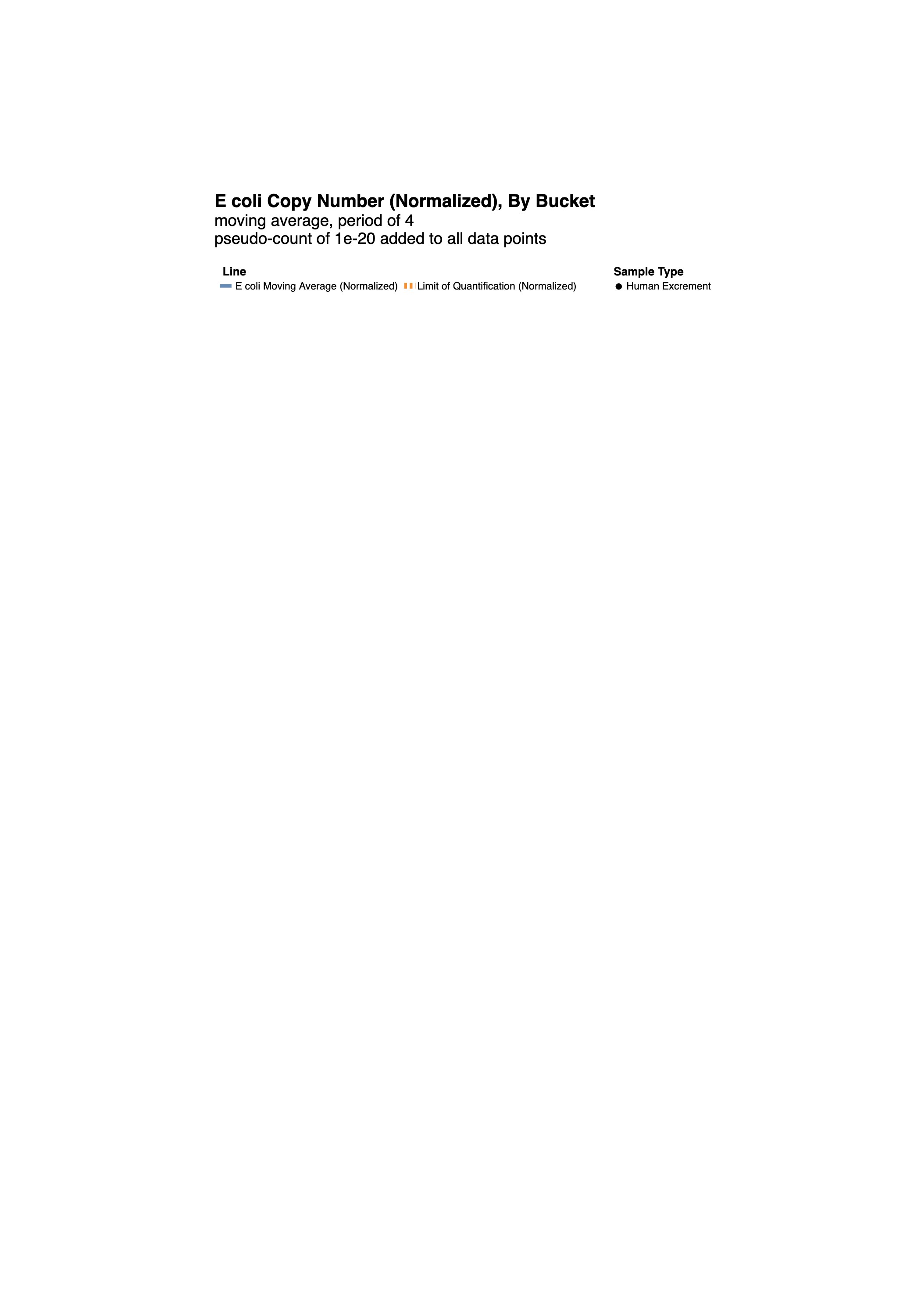

Supplement: figS4a-legend-ecoli_ycaf089 [file figs4a-legend-ecoli_ycaf089.jpeg]

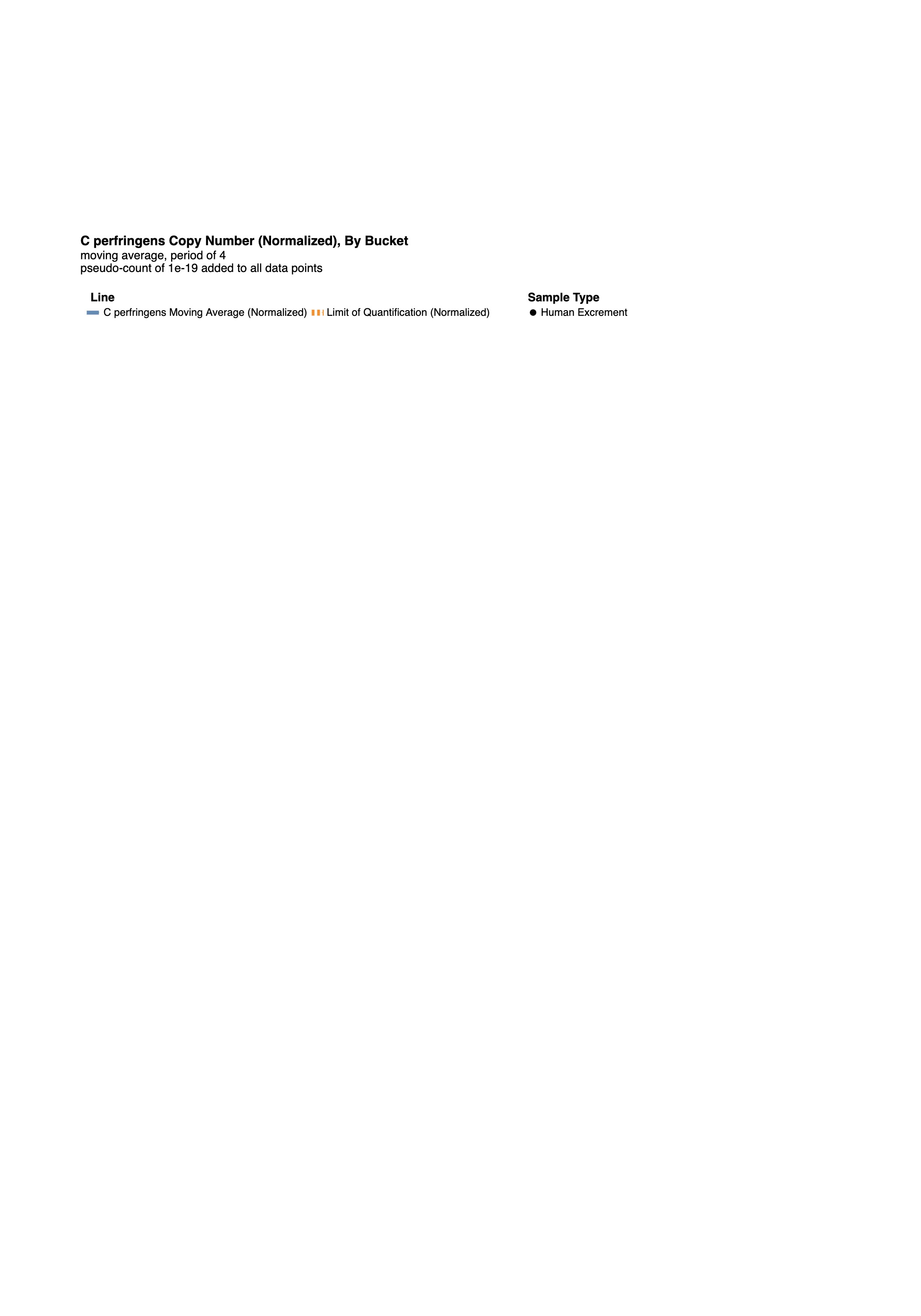

Supplement: figS4b-legend-cperf_ycaf089 [file figs4b-legend-cperf_ycaf089.jpeg]

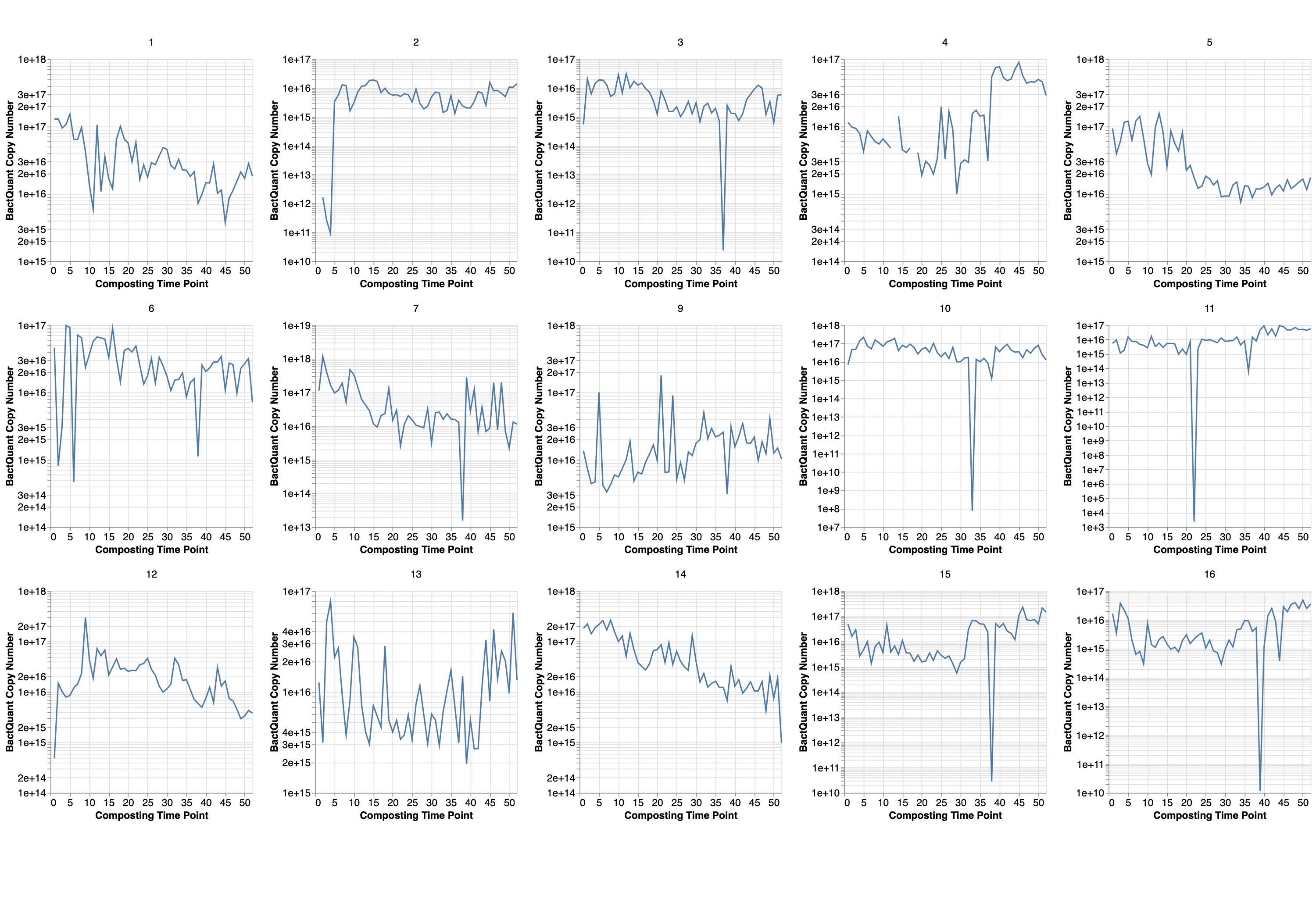

Supplement: figS5_ycaf089 [file figs5_ycaf089.jpeg]

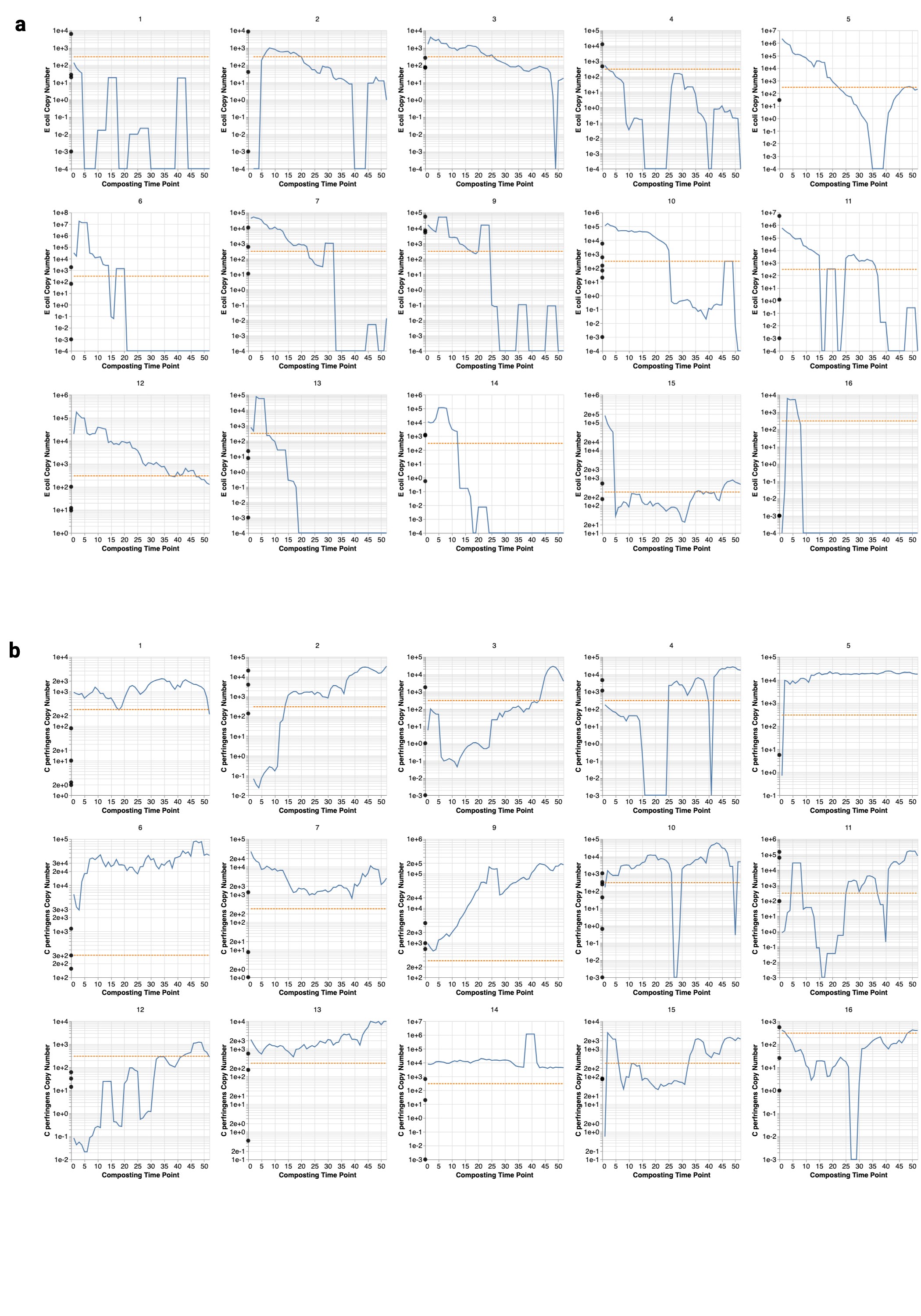

Supplement: figS6_ycaf089 [file figs6_ycaf089.jpeg]

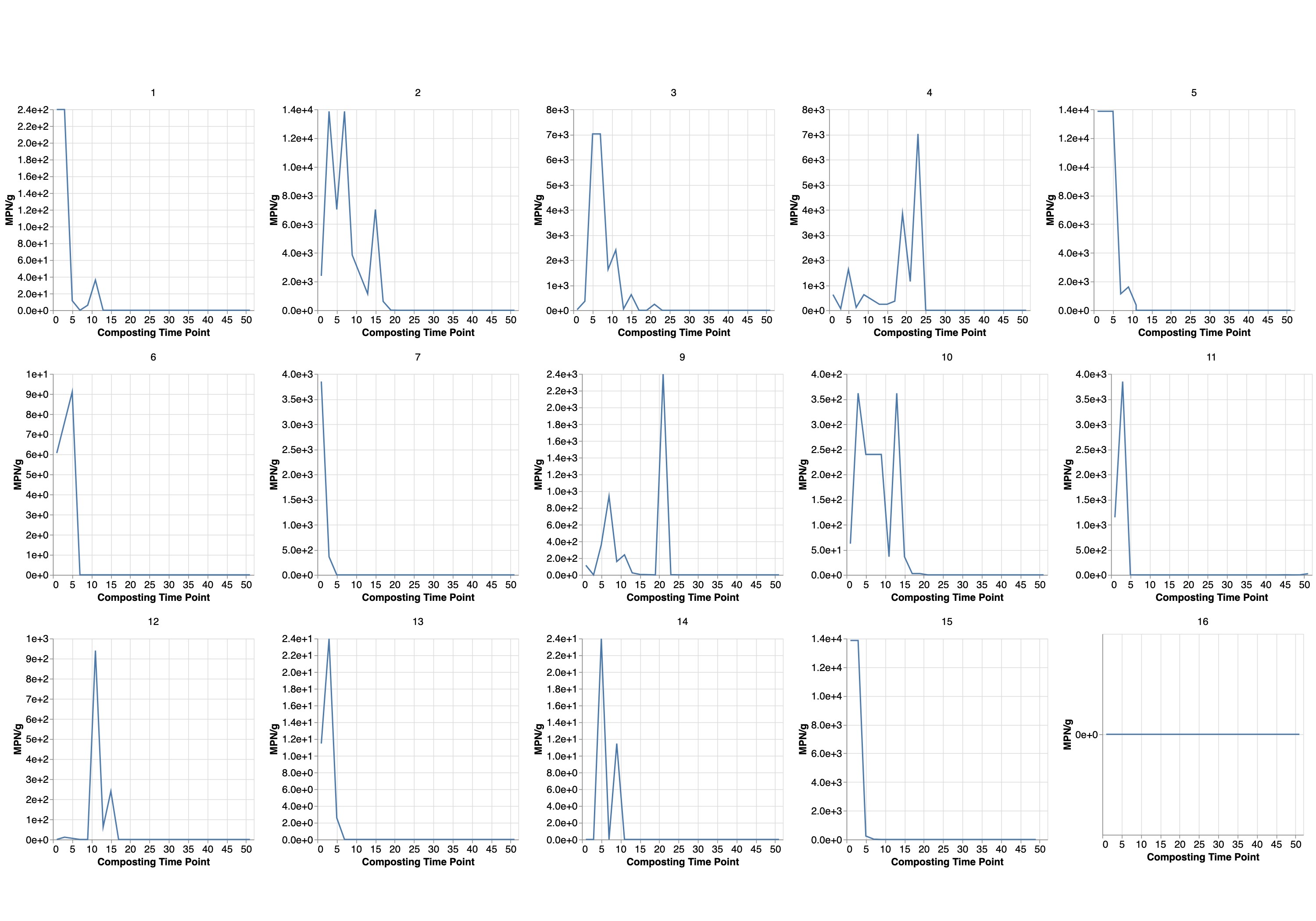

Supplement: figS7_ycaf089 [file figs7_ycaf089.jpeg]

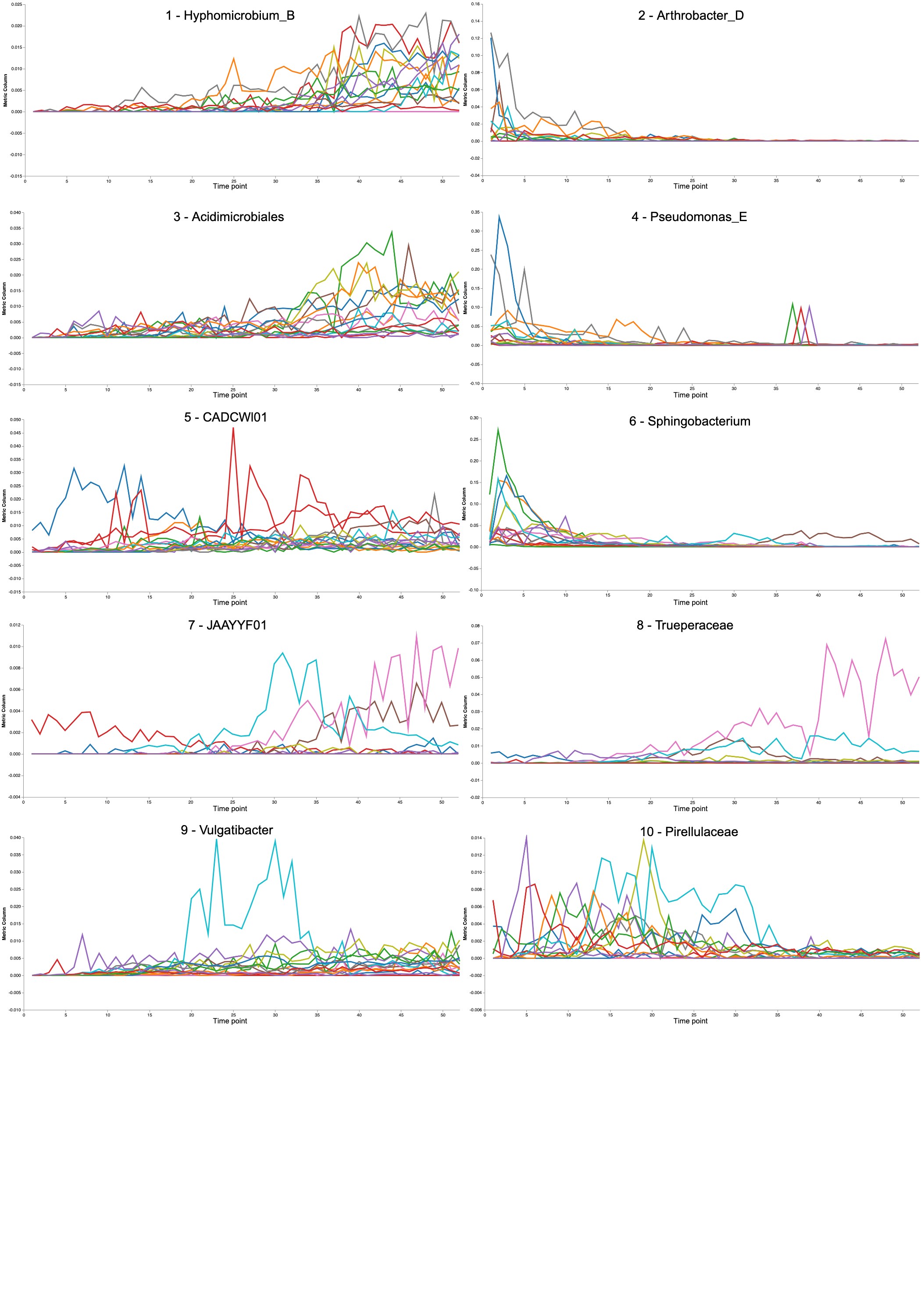

Supplement: figS8_ycaf089 [file figs8_ycaf089.jpeg]

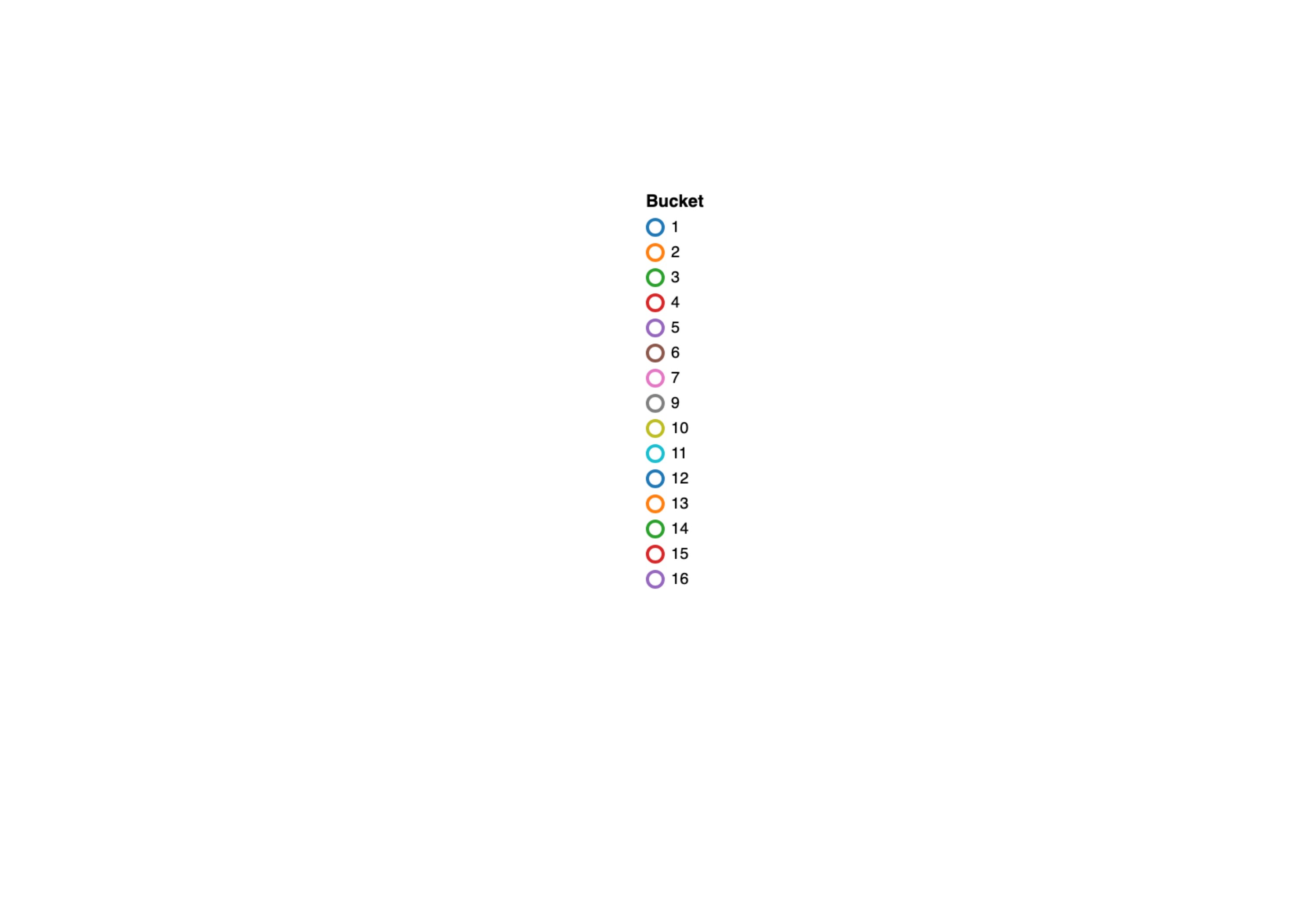

Supplement: figS8legend_ycaf089 [file figs8legend_ycaf089.jpeg]

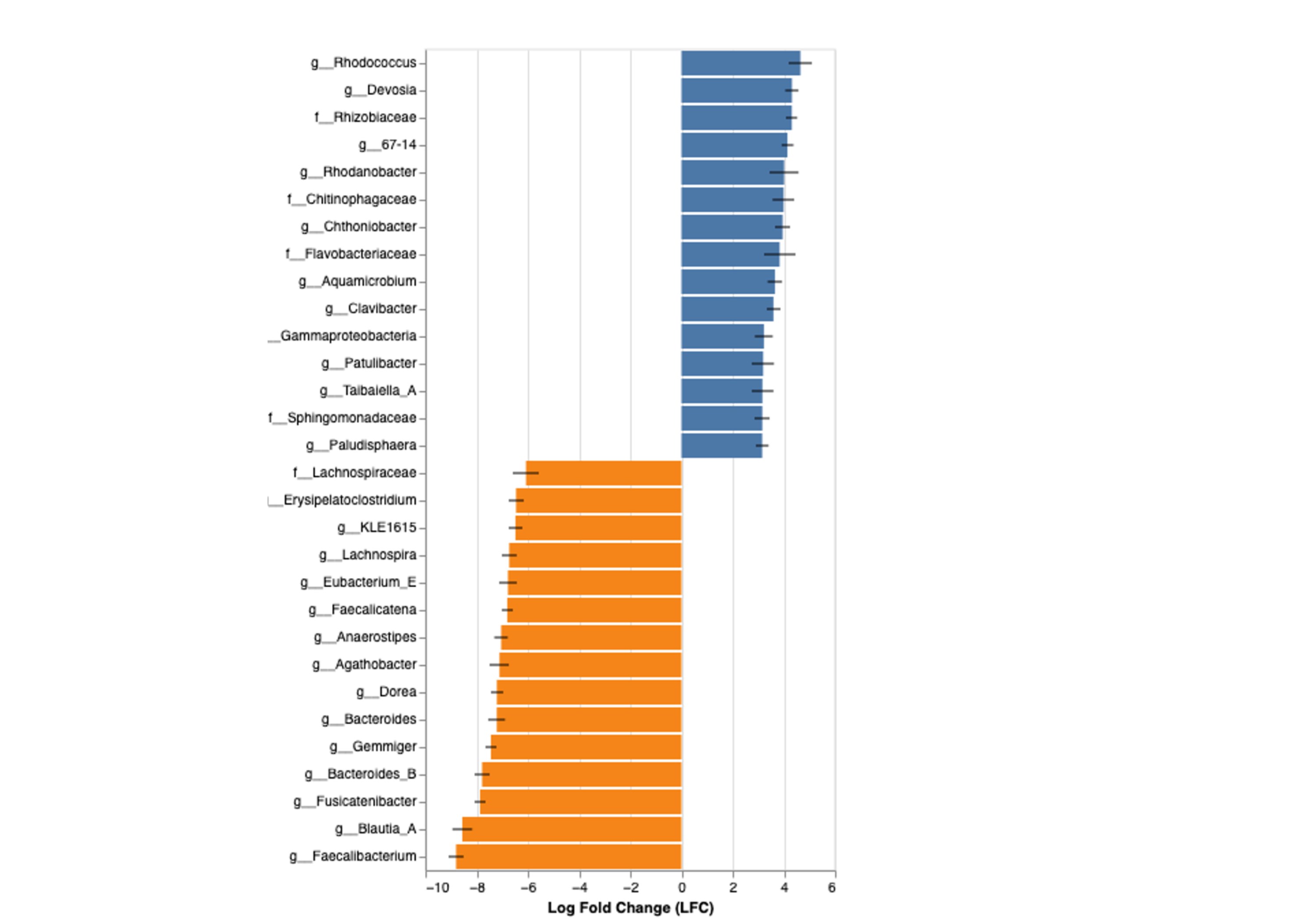

Supplement: figS9_ycaf089 [file figs9_ycaf089.jpeg]

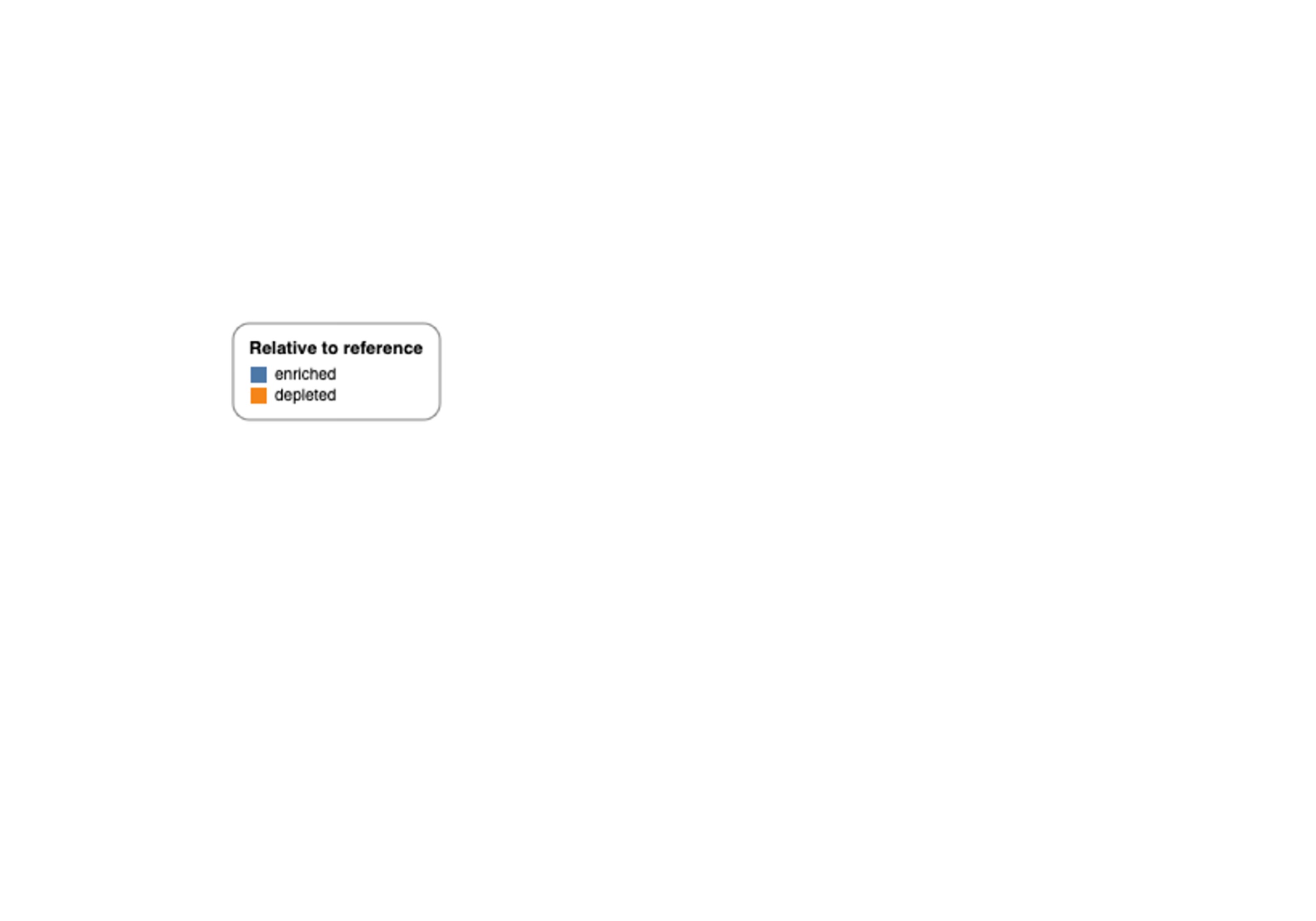

Supplement: figS9legend_ycaf089 [file figs9legend_ycaf089.jpeg]

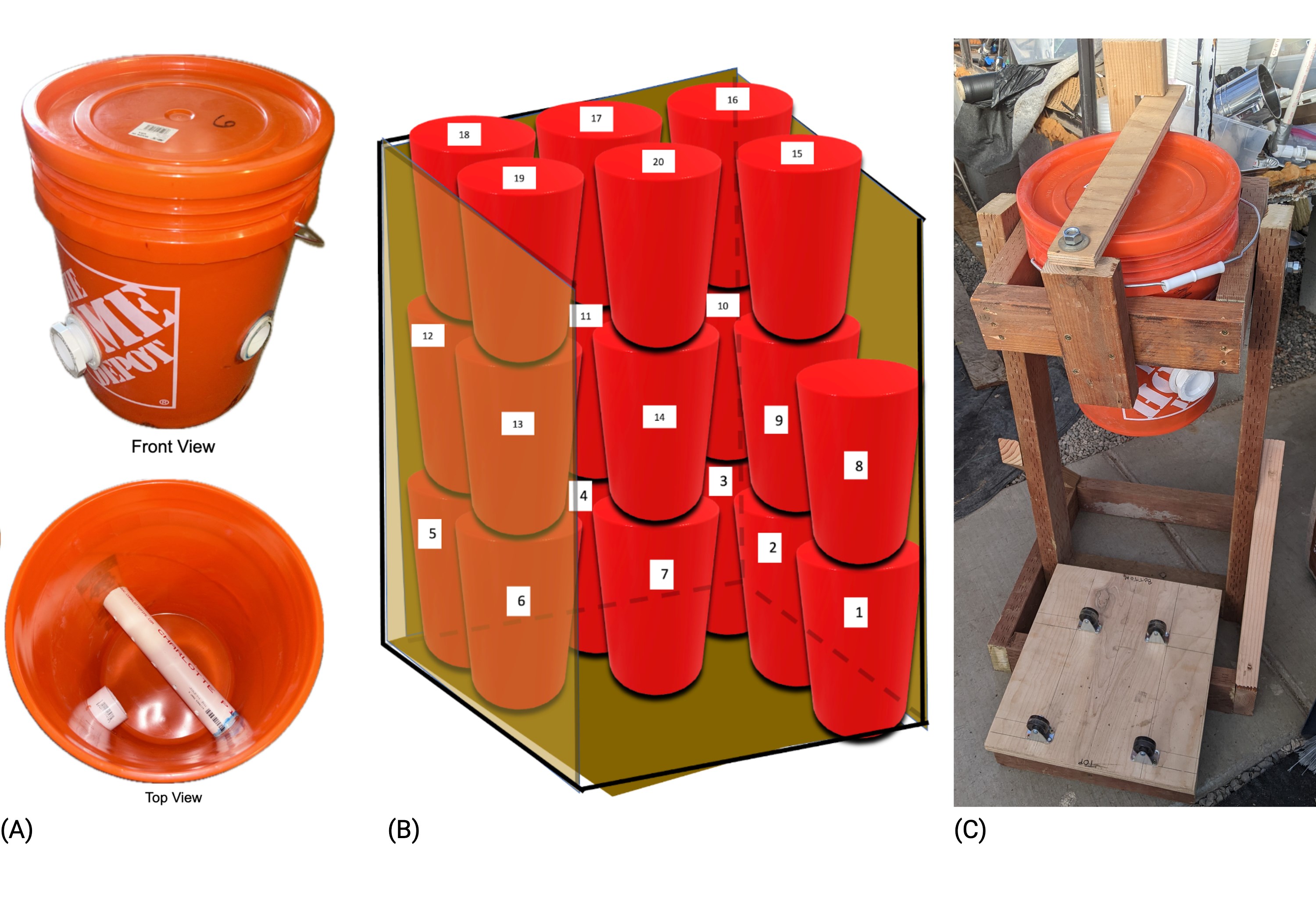

Supplement: figS10 [file figs10.jpeg]

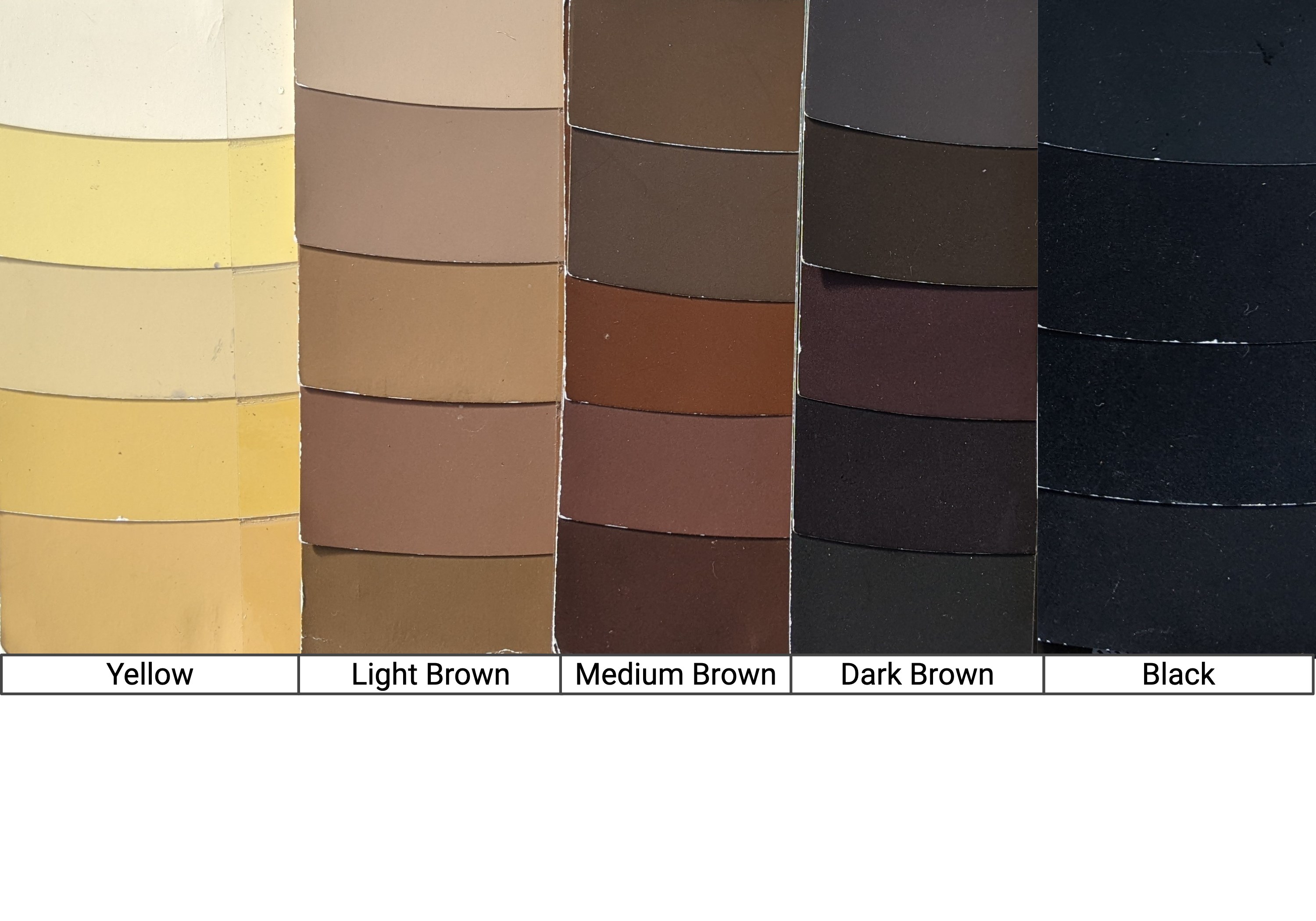

Supplement: figS11_ycaf089 [file figs11_ycaf089.jpeg]

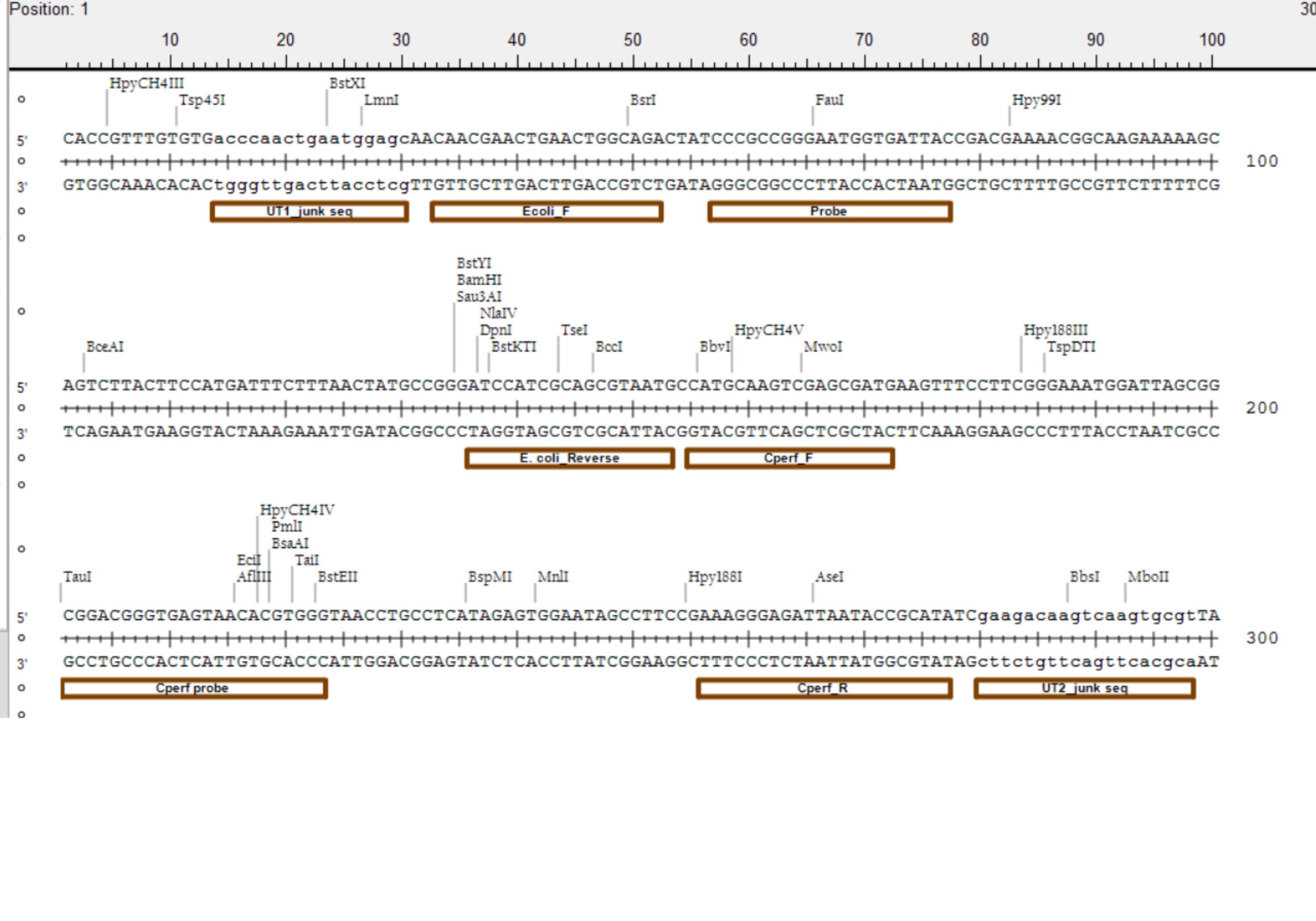

Supplement: figS12_ycaf089 [file figs12_ycaf089.jpeg]

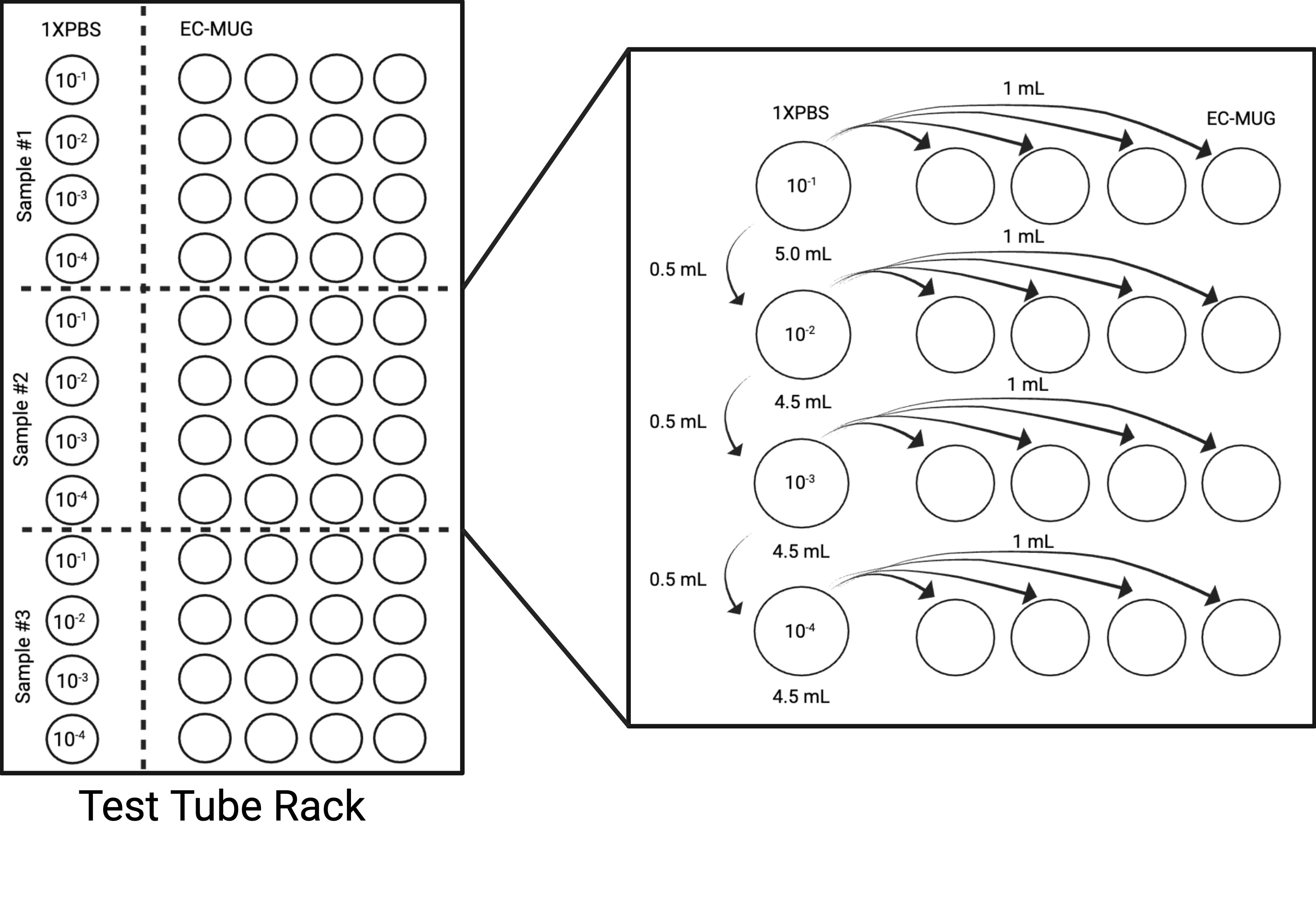

Supplement: figS13_ycaf089 [file figs13_ycaf089.jpeg]

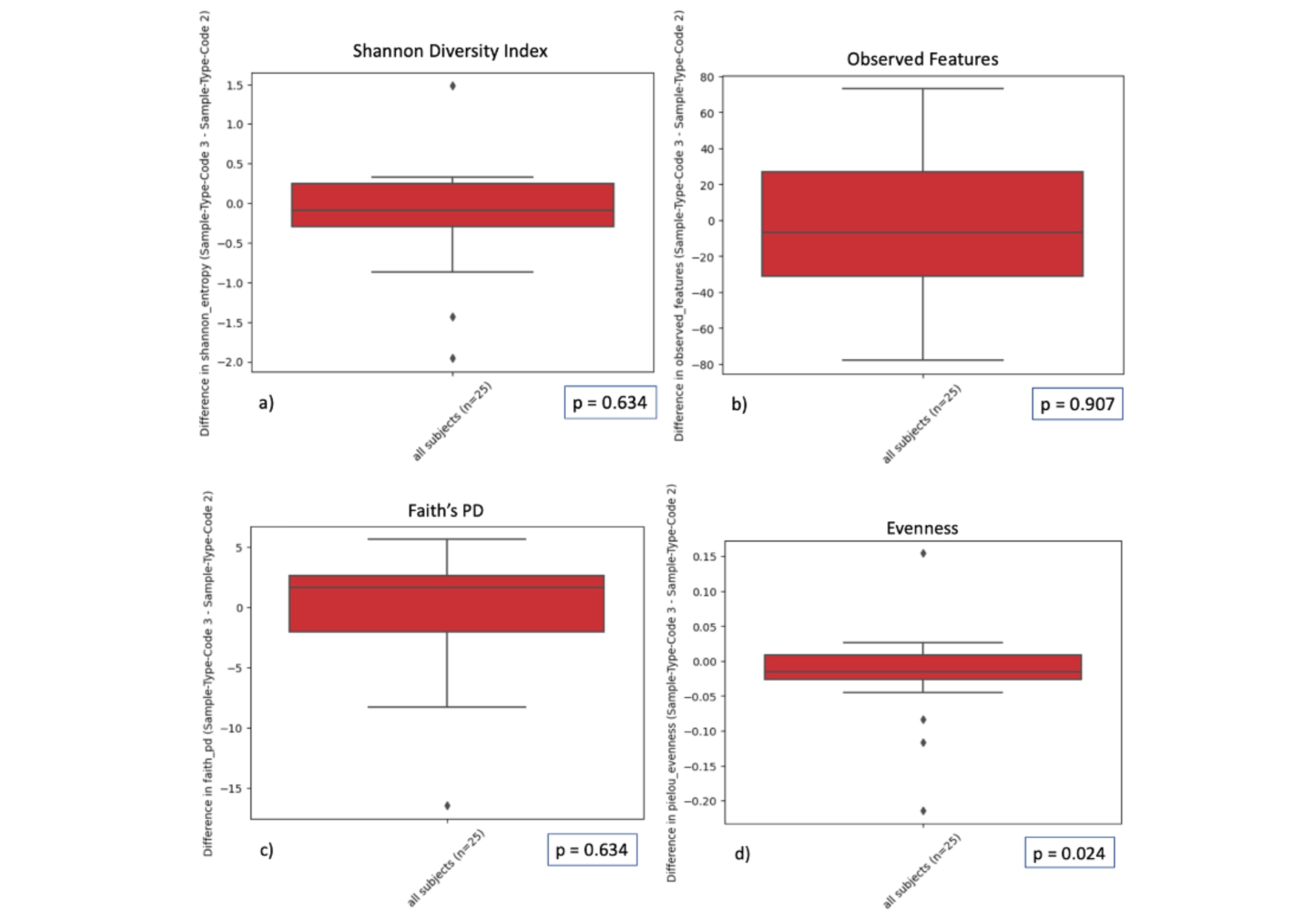

Supplement: figS14_ycaf089 [file figs14_ycaf089.jpeg]

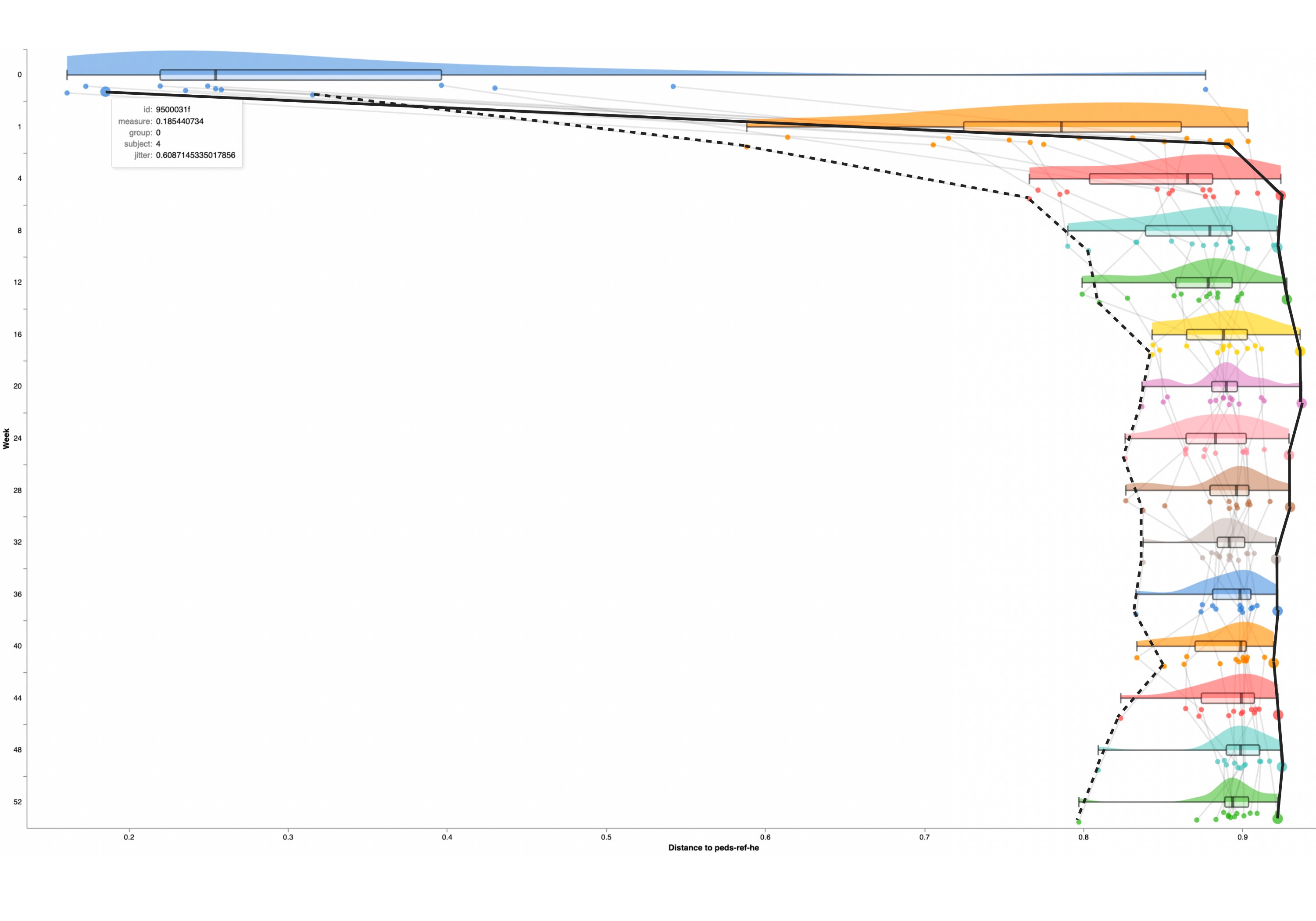

Supplement: figS15_ycaf089 [file figs15_ycaf089.jpeg]

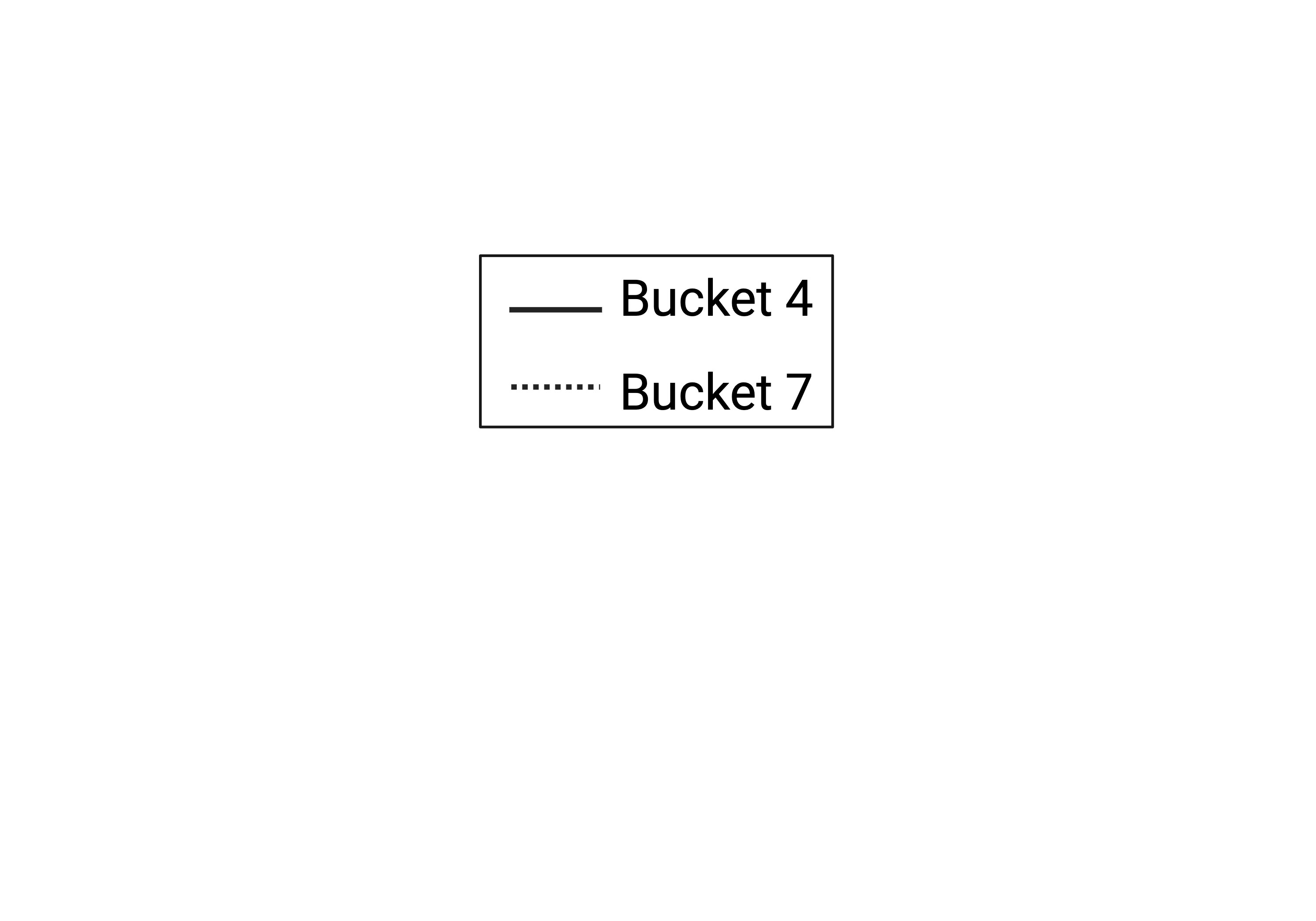

Supplement: figS15legend_ycaf089 [file figs15legend_ycaf089.jpeg]

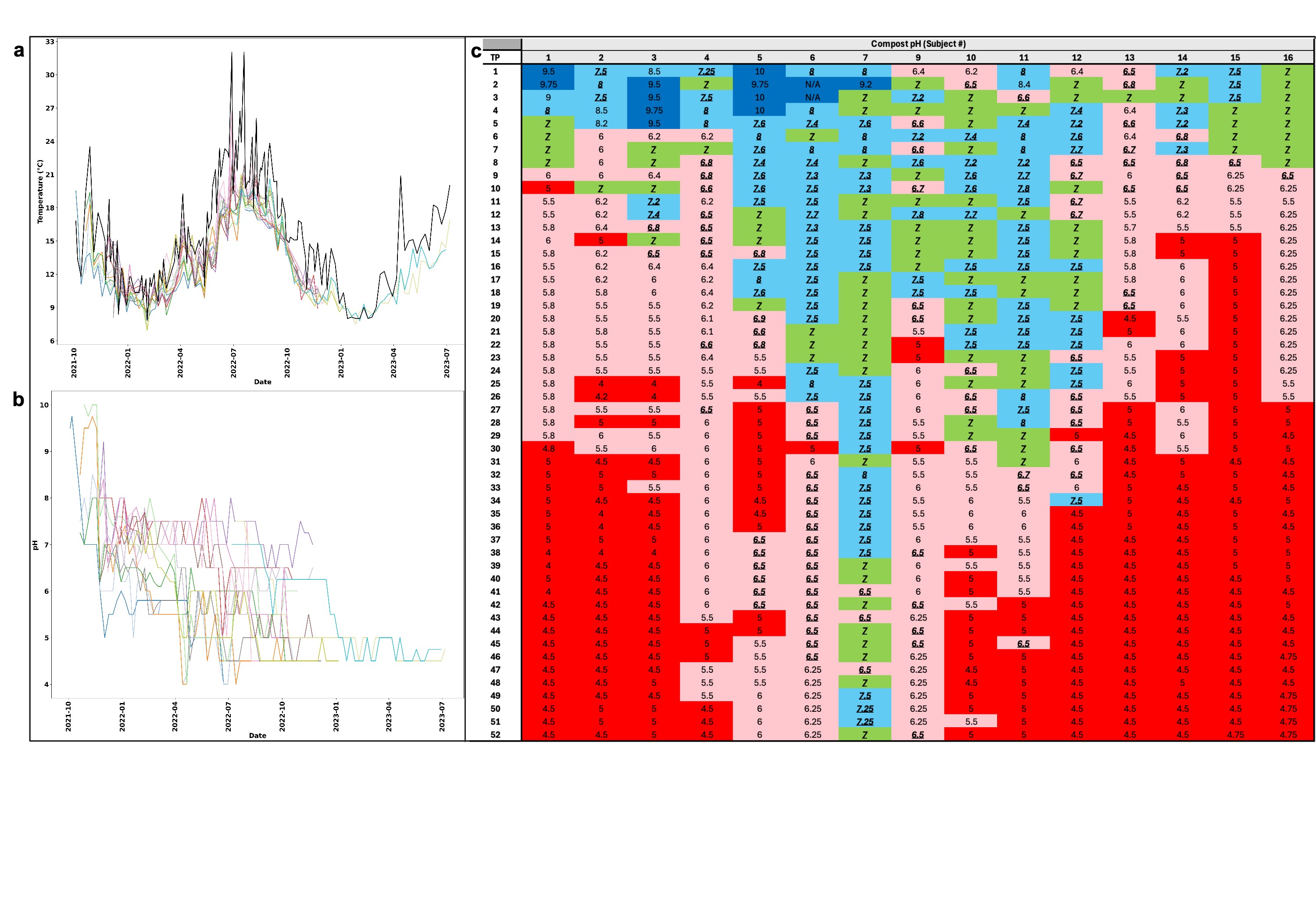

Supplement: figS16_ycaf089 [file figs16_ycaf089.jpeg]

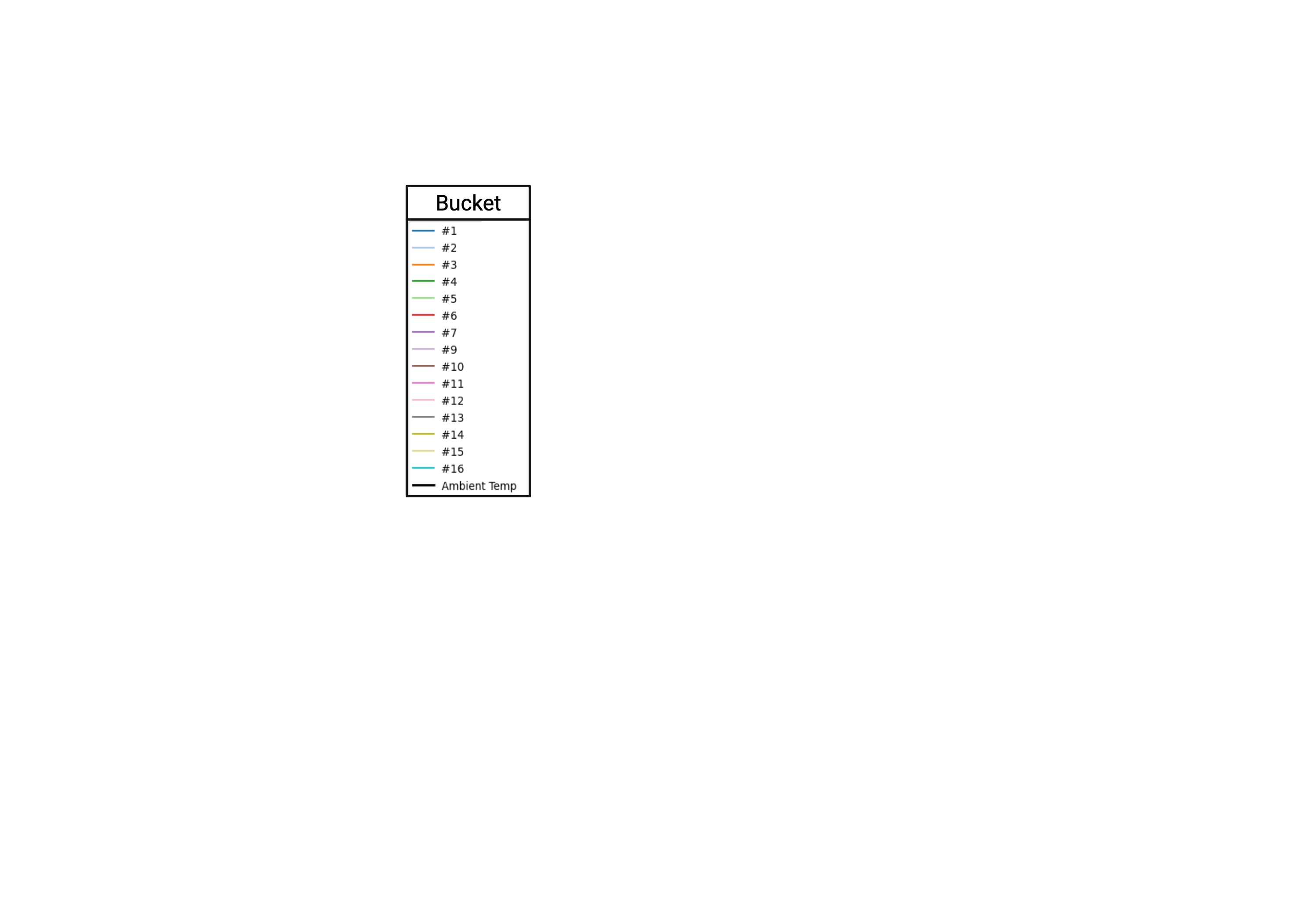

Supplement: figS16a-S16b-legend_ycaf089 [file figs16a-s16b-legend_ycaf089.jpeg]

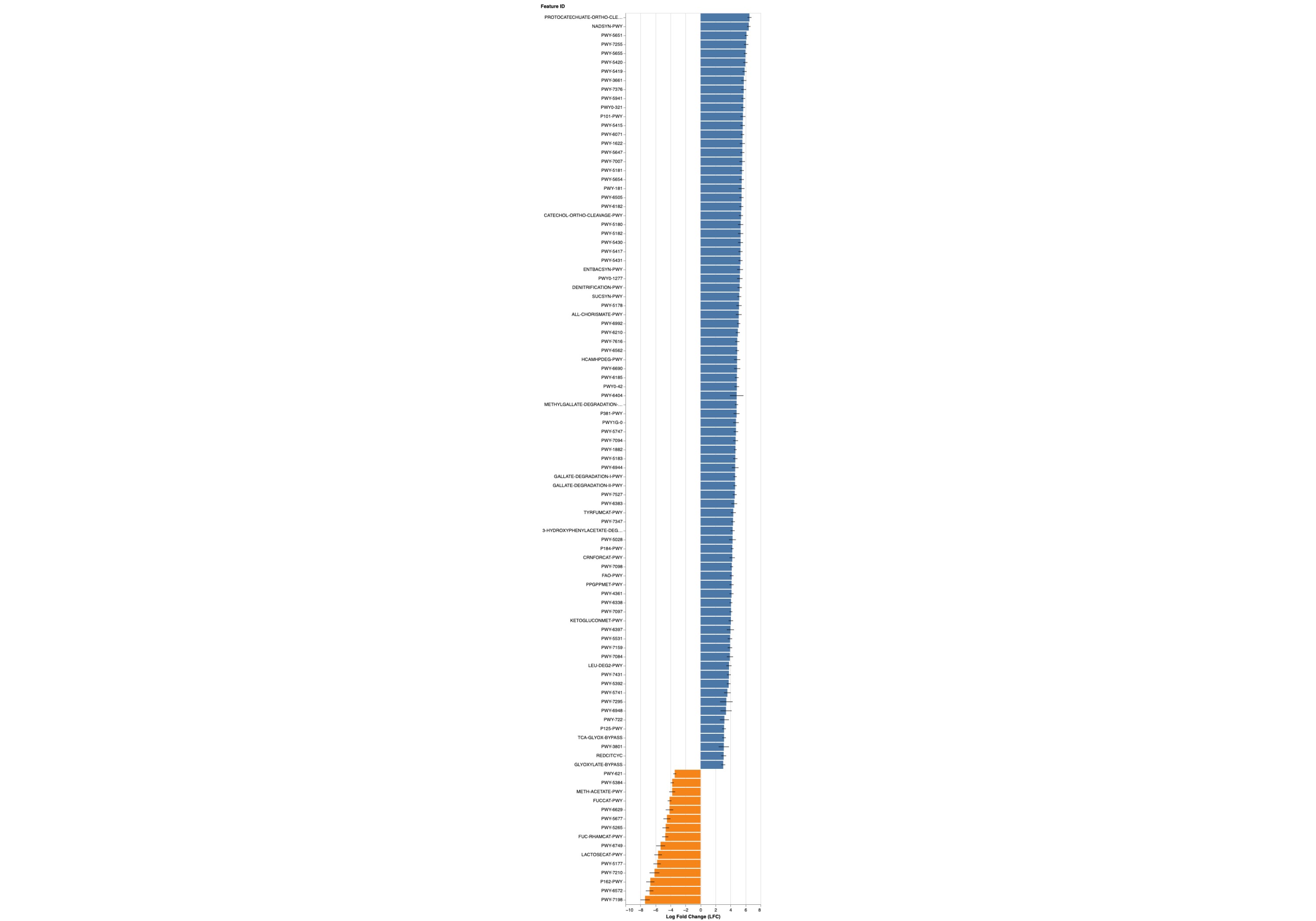

Supplement: figS17_ycaf089 [file figs17_ycaf089.jpeg]

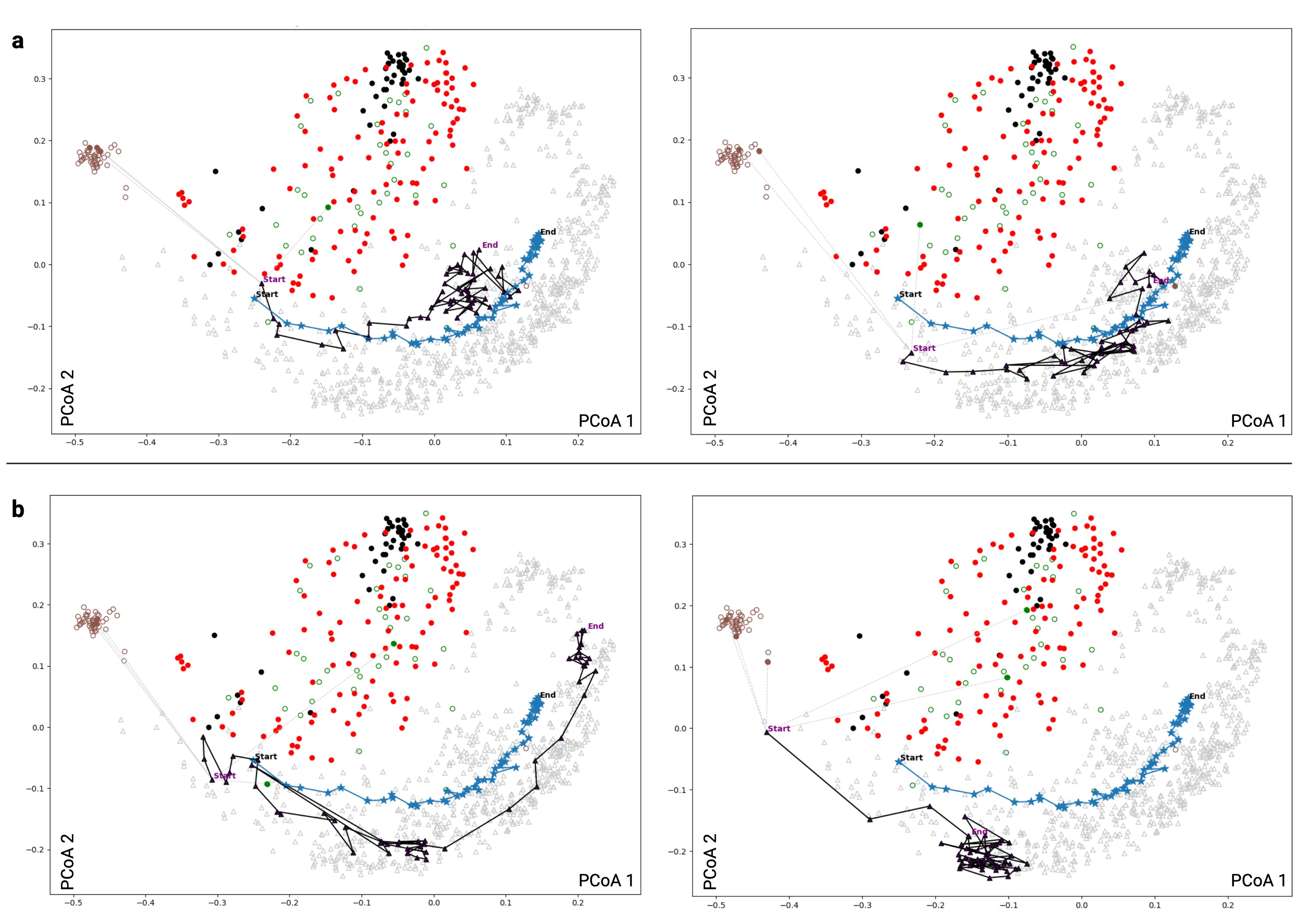

Supplement: figS18_ycaf089 [file figs18_ycaf089.jpeg]

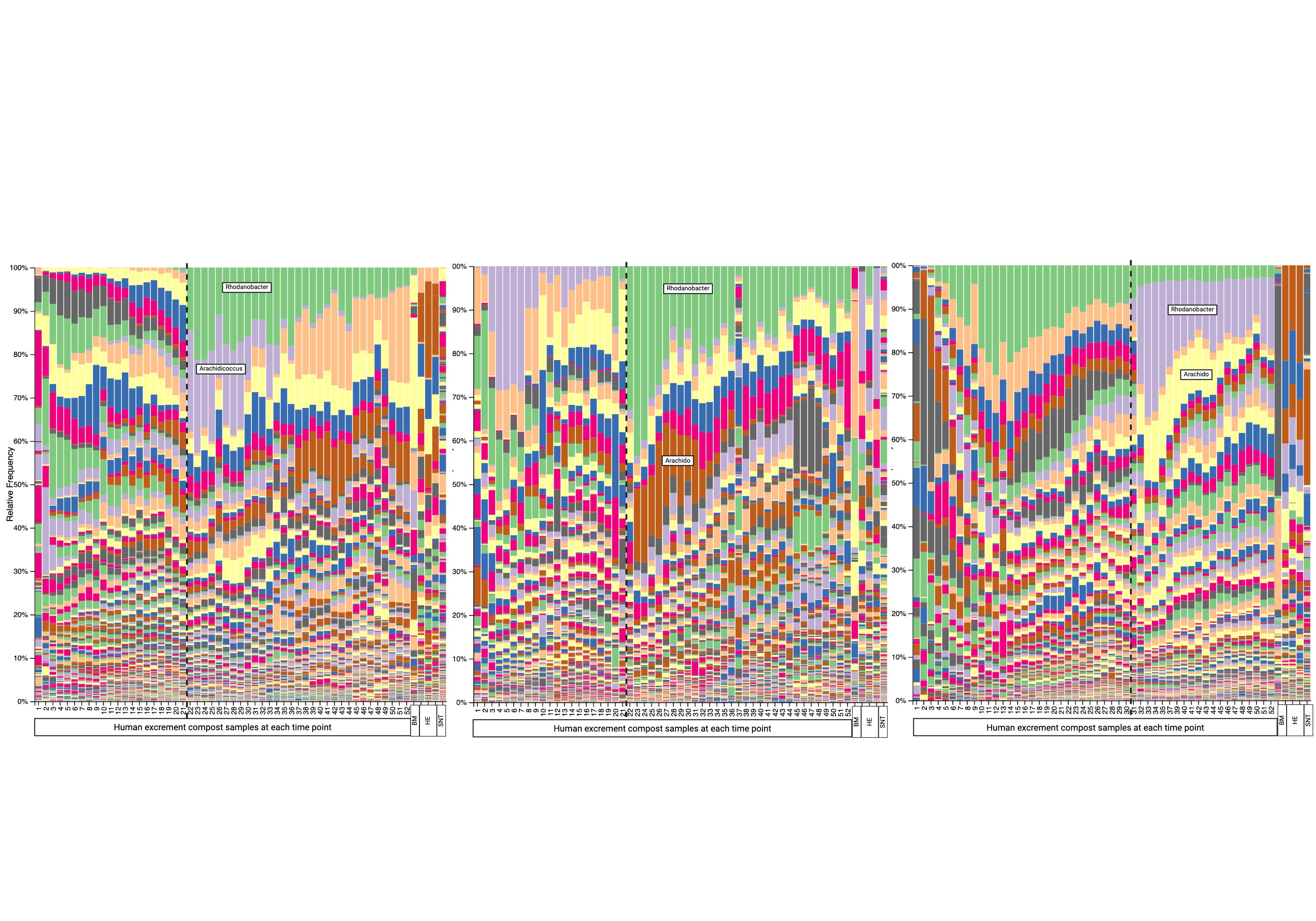

Supplement: figS19_ycaf089 [file figs19_ycaf089.jpeg]
